# Communicating oscillatory networks: frequency domain analysis

**DOI:** 10.1186/1752-0509-5-203

**Published:** 2011-12-22

**Authors:** Adaoha EC Ihekwaba, Sean Sedwards

**Affiliations:** 1 Dept. of Clinical Neurosciences Cambridge Centre for Brain Repair University of Cambridge, UK; 2 INRIA Rennes - Bretagne Atlantique Campus Universitaire de Beaulieu 35042 Rennes Cedex, France; 3 The Microsoft Research - University of Trento Centre for Computational and Systems Biology Trento, Italy

## Abstract

**Background:**

Constructing predictive dynamic models of interacting signalling networks remains one of the great challenges facing systems biology. While detailed dynamical data exists about individual pathways, the task of combining such data without further lengthy experimentation is highly nontrivial. The communicating links between pathways, implicitly assumed to be unimportant and thus excluded, are precisely what become important in the larger system and must be reinstated. To maintain the delicate phase relationships between signals, signalling networks demand accurate dynamical parameters, but parameters optimised in isolation and under varying conditions are unlikely to remain optimal when combined. The computational burden of estimating parameters increases exponentially with increasing system size, so it is crucial to find precise and efficient ways of measuring the behaviour of systems, in order to re-use existing work.

**Results:**

Motivated by the above, we present a new frequency domain-based systematic analysis technique that attempts to address the challenge of network assembly by defining a rigorous means to quantify the behaviour of stochastic systems. As our focus we construct a novel coupled oscillatory model of p53, NF-kB and the mammalian cell cycle, based on recent experimentally verified mathematical models. Informed by online databases of protein networks and interactions, we distilled their key elements into simplified models containing the most significant parts. Having coupled these systems, we constructed stochastic models for use in our frequency domain analysis. We used our new technique to investigate the crosstalk between the components of our model and measure the efficacy of certain network-based heuristic measures.

**Conclusions:**

We find that the interactions between the networks we study are highly complex and not intuitive: (i) points of maximum perturbation do not necessarily correspond to points of maximum proximity to influence; (ii) increased coupling strength does not necessarily increase perturbation; (iii) different perturbations do not necessarily sum and (iv) overall, susceptibility to perturbation is amplitude and frequency dependent and cannot easily be predicted by heuristic measures.

Our methodology is particularly relevant for oscillatory systems, though not limited to these, and is most revealing when applied to the results of stochastic simulation. The technique is able to characterise precisely the distance in behaviour between different models, different systems and different parts within the same system. It can also measure the difference between different simulation algorithms used on the same system and can be used to inform the choice of dynamic parameters. By measuring crosstalk between subsystems it can also indicate mechanisms by which such systems may be controlled in experiments and therapeutics. We have thus found our technique of frequency domain analysis to be a valuable benchmark systems-biological tool.

## Background

### Introduction

Many problems related to systems biology remain computationally hard (their difficulty increases exponentially with instance size), meaning that a brute force computational approach will only be tractable for small instance sizes. Despite apparently ever-increasing available computational power, in order to take full advantage of computational methods it is still necessary to apply them judiciously. This means balancing the requirements of precision and accuracy and finding meaningful abstractions which optimise them.

Representing signalling networks as dynamical systems of interacting populations of molecules offers the tantalising prospect of being able to predict the future behaviour of such networks by simulation. Precision in the *model *is high, but such systems are critically dependent on the accuracy of their parameters to produce valid predictions of *reality*. For relatively small subsystems ('pathways') it has nevertheless been possible to construct mathematical models that adequately reproduce the results of experiments, thus validating the model. With many such dynamically modelled pathways available in the literature and databases, it thus seems plausible to combine them into larger models able to better predict the behaviour of the whole system. This turns out to be not so easy. Dynamical parameters optimised in isolation and with respect to a particular set of experimental conditions will not necessarily be optimal in combination with other pathways. Moreover, the assumptions of external substances in excess or at equilibrium used to mathematically isolate the model may mask complex mechanisms as yet unmodelled. Thus, blindly re-connecting the disparate systems is not valid and experimental validation of every combination of pathways is impractical.

In order to take advantage of the vast repository of accumulated data and the easy availability of computational power, we have devised an efficient systematic approach that allows automatic analysis and verification of large dynamical models in a meaningful way. Noting that oscillatory behaviour is ubiquitous in biological systems, we present a new automated analysis technique based on frequency domain analysis, able to measure precisely the behaviour (oscillatory or otherwise) of interacting systems. To demonstrate the utility of this approach we apply it to a novel coupled oscillatory model of p53, NF-kB and the mammalian cell cycle. In what follows we first describe the background to the modelling process and explain our methodology in detail, we then present and discuss our results and finally draw conclusions. An additional file contains further background to the modelling and analysis process, plus detailed descriptions of the models we have created.

### Biological context

It is well-known that signalling pathways that govern cellular death are of critical importance for normal tissue development, homeostasis and function [1,2]. Many pathological implications are associated with dysregulation of the delicate balance between cell life and death. In mammalian cells, various signals, such as hormones, cytokines, and cell-cell interfaces, elicit changes at the gene expression levels, mediated by inducible transcription factors that provide feedback loops upon their signalling pathways. These feedback genes, generally thought to functionally terminate the signalling action of the transcription factor, create the potential for the transcription factor activity to oscillate between active and inactive states over a period of hours [3-6].

Oscillations are necessarily ubiquitous in biology and are found, for example, in the pulse of the heart, the circadian rhythm, in the signal transduction that involves adenosine 3',5'-cyclic monophospate (cAMP) and in the chemotaxis of *Dictyostelium discoideum *[7]. In the present context it is important to note that oscillatory behaviour is evident in the cell cycle, nuclear factor-κB (NF-κB [3,8-10]) and p53 [4,11,12]). However, the precise significance of all such oscillations is still unclear; how the cell uses oscillations to differentiate input conditions and send specific signals to downstream genes have been central questions in the study of signalling pathways. This strongly motivates the need for an engineering approach to quantify these effects in biological systems exhibiting emergent oscillation.

In the literature, qualitative descriptions of the components and mechanisms of oscillatory signalling systems have greatly improved our understanding of how cells function and have given insights into their behavioural properties, along with how to intervene therapeutically when such signals are mis-communicated [13-15]. Theoretical studies have shown that many important biological effects can be adequately modelled as simple processes of information transfer on top of assumptions of equilibrium concentrations of metabolites and thus pathways have been successfully examined in this fashion [13-15]. In reality, the architecture of signalling pathways is much more complex, involving time, space and frequency. To account for the complex, multi-dimensional behaviour now observed in experiments, some simplifying assumptions (such as equilibrium) can no longer be treated as valid and a greater level of complexity must be considered [16-21]. It is this paradigm shift and the demand for increased fidelity and predictive accuracy of models that makes understanding signalling in general a challenging task and that have made it necessary to include the many non-linearities present in reality.

### Technical motivation

Full mathematical analysis of interesting biological systems is usually impractical; the simplifications that are effective for small systems are generally not scalable. Moreover, low dimensional explanations of highly complex behaviour seem to defeat the purpose of constructing large models. For large systems we require a *systematic *approach, so here we present an automated analysis technique based on Fourier transformation of simulation traces. By transforming the time series produced by stochastic simulations into the frequency domain, it is possible to characterise mathematically the behaviour of both oscillatory and non-oscillatory systems over time. The use of stochastic models is motivated by the presumption that the underlying mechanism of molecular interactions is discrete and that such models therefore more accurately represent reality. As a consequence, our technique reveals more information about the system than may be possible to extract from deterministic simulations (i.e., the numerical solution of differential equations); *variance *plays both qualitative and quantitative roles. Improved computer hardware and the development of simulation algorithms has made stochastic simulation computationally viable: it is now usually possible to complete multiple parallel simulation runs of large systems in a matter of minutes. Taking advantage of this, we construct frequency spectra from multiple simulation runs in order to characterise the average simulated behaviour. In contrast to deterministic simulations, these spectra contain detailed (i.e., frequency and phase) information about variance. Measures over these spectra may then be used to quantify differences and similarities between different systems, different parts within a system, different models of the same system or different simulation algorithms etc. In the present investigation we use this technique to analyse the crosstalk between linked oscillatory systems and the effects of stochasticity. We do this by measuring the differences between combinations of the coupled and uncoupled systems and by measuring the differences between stochastic and *quasi*-deterministic models (see Methods). The component subsystems have different characteristic frequency 'signatures' that allow us to identify which system(s) are responsible for a particular perturbation, in addition to characterising its magnitude.

### The model

To demonstrate the ideas and power of the proposed method, we apply it to theoretical models of transcription factors identified to play critical roles in cell differentiation and cell death. Aberrant NF-κB (p50/p105, p52/p100, RelA, c-Rel, RelB), best known for its role in immune and inflammatory responses, is an active growth- and division-promoting transcription factor [22]. By contrast, the activation of the p53 transcription factor (a well-known tumour suppressor gene) in response to DNA damage and hypoxia, transcribes a series of genes that initiates cell cycle arrest, apoptosis or senescence, eliminating clones of cells with DNA damage and the resultant mutation. Thus the p53 response to its stress is the opposite of the NF-κB response to infections or cytokines. That is not to say that there is no overlap in the functions of NF-κB- and p53-regulated genes. Under appropriate stress signals the NF-κB have been shown to initiate programmed cell death [23,24], while p53 initiates the transcription of several cytokines [25]. In general, however, these two systems respond to stress signals using very different and often mutually exclusive transcriptional mechanisms [26,27].

We have extended the chosen models to include their involvement with the cell cycle. For example, an immune response to a foreign organism results in the promotion of the target gene cyclin D1; and a response to a high mutation or error rate brought about by DNA damage results in the transcriptional upregulation of target gene p21 via p53 to initiate cell cycle arrest. Cyclin D1 promotes cell cycle progression through G1-phase by forming active holoenzymes with CDK (cyclin-dependent kinase) 4 and CDK6. CDK4 and 6 phosphorylate the Rb (retinoblastoma protein) [5,28] and cause Rb to release the E2F transcription factor which can then activate genes essential for G1-S transition and S-phase [29]. By contrast, association of p21 with cyclin D-CDK4/6 inhibits Rb phosphorylation and induces cell cycle arrest in G1. Through its negative effects on various CDKs, p21 inhibits both the G1-to-S and the G2-to-mitosis transitions. p21 also associates with and inactivates E2F, leading to cell cycle arrest and cellular senescence. Considering the deregulation of NF-κB and p53 pathways, it is not surprising that an extensive crosstalk between both pathways exists at various levels [30].

## Methods

We are principally interested in the interactions of the processes generating oscillation, so our approach is to find simple models which nevertheless capture the fundamental characteristics of their oscillatory behaviour at a mechanistic level. We considered published mathematical models of the IkB-NF-kB [8,10,31-35], mammalian cell cycle [36] and p53-Mdm2 [4,11,37-40] systems that describe their evolution in time. Our aim was then to construct a simple, unified model that captures faithfully the important elements of the original systems, including stochasticity, thus facilitating efficient analysis and accurate predictions.

### Model creation

Models (networks) taken from the literature and databases often contain elements not crucial to the observed behaviour but included as the valid results of research and experiments. With judicious pruning (see e.g. [41] chapter 6), such elements may be safely removed; in addition to simplifying the task of simulating and analysing such networks, removing unimportant parts reduces the possibility of over-fitting experimental data when inferring dynamical parameters [42]. It is important to note, however, that such simplification is not a requirement of the frequency domain analysis we will present below. The computational cost of our technique tends to increase as a low order polynomial with respect to system size (see below), while the cost of model creation (including, e.g., parameter estimation) tends to scale exponentially. To generate our combined model, we reduced experimentally validated models of the individual pathways and linked them with plausible coupling reactions. Traditionally for the NF-kB pathway, removal of the other isoforms of the canonical IkB is a common simplification in computational analysis of the pathway [32,43,44], however it tends to overlook the fact that IkBα negative feedback alone exaggerates oscillations. To focus on the processes we were interested in, models from [8,34] and [35] were chosen as our starting point, with some specific parameter changes: rate values for relevant reactions involved in the creation or destruction of the IkB isoforms were averaged or summed (as applicable to the parameters being changed), so that only one IkB isoform was utilized in the end. Since the inhibitors have been shown to maintain the dynamic - oscillatory - behaviour observed for the NF-kB pathway [10,31,34,45,46], all rate equations governing their reactions have been taken into consideration as the system is reduced. Since current knowledge of the p53 system is incomplete, we analysed the simplest consistent model, combining features of [11] and [40], where the assumption is that a protein downstream of p53 inhibits a signalling protein that is upstream of p53 (elements of which could be, e.g., phosphorylated ATM) which may or may not undergo oscillatory dynamics. This assumption was inspired by the observation that phosphorylated ATM, an upstream regulator of p53 [47], responded to double-stranded DNA breaks (DSBs), showing a pulse of activity [48,49]. The model (VI of [4]) uses two negative feedback loops, one direct feedback and one longer loop that impinges on an upstream regulator of p53.

For their involvement with the cell cycle, the two pathways were connected via components whose regulation is activated by one pathway but coupled to substrates belonging to the G1/S phase of the cell cycle network. Such components are the promoter activity of cyclin D1 molecules (a protein required for cell cycle progression from the G1 phase to S phase) that have been shown to be activated by NF-kB transcription factor [50,51];, the p21 molecules (an inhibitor of the G1/S progression protein) activated by p53 molecules, and finally the p14-ARF (a cell cycle protein) known to inhibit Mdm2 activity.

### Stochastic modelling

In designing the linked systems, both deterministic and stochastic methods were utilized. Up-to-date models were taken from the literature in the form of ordinary and delay differential equations. Links were hypothesised based on a literature search and the models were simplified and parameterised using the assumptions outlined above and in Additional file 1. To validate our simplifications, deterministic simulations were performed to verify that the key behavioural characteristics of amplitude and period of oscillation were consistent (better than ± 5%) with those of the original, experimentally verified, models. Further simulations were performed to verify that the behaviour of the coupled models was equally consistent. The models were then converted into quasi-deterministic and fully stochastic forms for simulation using the method of arbitrary partial propensities (MAPP [52]), an 'exact' variant of the Gillespie direct method [53]. In the case of the quasi-deterministic models, the transformation is essentially a conversion of the ordinary differential equations (ODE) from continuous concentrations into discrete numbers of molecules. Although in theory our frequency domain analysis also works with fully deterministic simulations, deterministic spectra contain no information about variance (so are uninteresting from our point of view) and often contain arbitrary artefacts arising from the practical limits of numerical precision (ODEs assume infinite precision) and the adaptive nature (variable internal time steps) of numerical solvers. The inherently 'spiky' nature of these spectra potentially make measurements more fragile in comparison to those of stochastic spectra. Additional file 1 Figure S7B illustrates the spikiness of a deterministic spectrum and its relationship to non-deterministic spectra of corresponding stochastic and quasi-deterministic models. To discretise both the quasi-deterministic and stochastic models the initial concentrations were multiplied by a constant (denoted *alpha*) having units of *l mol*^-1 ^that was also used to transform the rate constants (see Additional file 1 supplementary methods for details). To create the fully stochastic models, the terms of the differential equations were separated to form elemental reactions of the form A + B → C + D, using mass action kinetics. Note that three of the reactions of the p53 system, one of the NF-κB system and the coupling between the NF-κB and the cell cycle systems employ kinetics that are not mass action and are converted to reactions with parameters that respect their specific kinetic functions. While it may be desirable to reduce the entire system to elemental reactions in order to preserve the physical assumptions made by the stochastic simulation algorithm [53], this is not necessary from the point of view of our analysis. Indeed, the questions raised by not using elemental reactions may be answered by our technique and motivates the inclusion here of quasi-deterministic versions of our models. It is important to note, however, that our conversion procedure guarantees that for *any *specified initial state, the instantaneous magnitude and direction of the average rate of leaving the state in the stochastic and quasi-deterministic models is *identical *(allowing for the change from concentration to numbers of molecules) to that of the deterministic model. The subsequent traces would, of course, be different, but by maintaining *local *consistency we are justified in re-using the dynamical parameters of the original models.

### Stochastic simulation

Simulation is a very simple means to get an idea of the behaviour of a dynamical system. In a deterministic framework the evolution of concentration in time produced by numerically solving a set of ODEs is a direct characterisation of its average behaviour, but individual stochastic simulation traces may be quite different from one another. There is often an intuitive notion of average behaviour, apparently related to the solution of the corresponding ODE, but this is merely coincidental. Since such an ODE defines the behaviour of the stochastic system taken to the *thermodynamic limit *[54], it is not in general the average of the stochastic process. Importantly, the noise in stochastic simulations is not merely superimposed on an underlying deterministic trajectory, but is created by the mechanism of the system and is therefore *intrinsic *to the trajectory. Additional file 1 Figure S7B illustrates the significant differences between deterministic and stochastic models constructed from the same reactions and kinetic parameters.

The stochastic models we consider here are governed by the chemical master equation (CME, see e.g. [54]), which is a linear differential equation that describes the evolution in time of the probability of the system being in any particular state, considering *all *possible evolutions from the initial state. It is possible to solve the CME numerically and thus obtain the distribution of values that a molecular species may assume at a given time point. Such a distribution is with respect to all evolutions and does not consider how an individual trajectory may have arrived at a particular value (there will likely be multiple routes, via multiple sequences of reactions). Causality is lost. Solving the CME is therefore not useful in describing oscillatory behaviour: neither the oscillations nor their properties may be evident in the resultant distributions.

Thus, while the choice of a discrete stochastic framework offers the potential to investigate chemically reacting biological systems in the most precise way, in order to draw general conclusions about a model's behaviour from stochastic simulations it is necessary to characterise some kind of average trajectory that preserves the behaviour. Averaging the time series of multiple stochastic simulation runs, however, does not produce an average trajectory: the amount of a molecular species at a given time point in different simulation runs is a random variable, the distribution of which being defined by the CME. The consequence of this is that averaged oscillatory behaviour of stochastic time series tends to disappear with increasing time because as time progresses the system is less likely to be in a unique state. This is illustrated in Additional file 1 Figure S7A, where it is clear that behavioural information is progressively lost to the averaging process. By considering the *average frequency spectra*, however, we avoid this limitation and can take full advantage of the information contained in the stochastic traces.

### Statistical measures over frequency spectra

We make multiple simulation runs (100 for the presented results), having identical initial conditions and length of simulated time, and the resulting time series are converted to complex frequency spectra using the discrete Fourier transformation (DFT):

(1) $$f_{\omega }=\overset{N-1}{\underset{n=0}{\sum }}x_{n}e^{-\frac{2i\omega n}{N}}$$

*f_ω _*is the *ω*^th ^frequency component (of a total of *N*) and *x_n _*is the *n*^th ^(of *N*) time sample of a given molecular species. In practice, this will be achieved efficiently by using a standard Fast Fourier Transform (FFT) algorithm. Stochastic simulations resulting from a variant of the Gillespie algorithm [53], as used in our investigations (MAPP [52]), produce time series having irregular time spacing between points. Hence, to apply Equation (1), which assumes constant time steps, it is necessary to sample the stochastic time series at regular time intervals. The method adopted is to calculate *x_n _*= *x_t _*| max(*t *≤ *n δt*), where *x_t _*is the simulation point having value *x *at time *t *and *δt *is the desired sampling time step. Intuitively, this formula gives the last value recorded prior or equal to the required sample time. The combination of *N *and *δt *define the overall time that the system is observed (*Nδt*), the frequency resolution ((*Nδt*)^-1^), and the maximum observable frequency ((2*δt*)^-1^). To maximise the range and the precision of the analysis it is generally desirable to have large *N *and small *δt*, however these must be optimised with respect to the phenomena being investigated; in addition to the computational cost of excessive range and precision, there may also be an unforeseen loss of resolution. A reasonable lower bound of *δt *might seem to be the time of the shortest individual reaction event found in the time courses, however this is often excessively short, extending the frequency spectrum orders of magnitude above the interesting phenomena. Similarly, lengthening the overall simulation time, thus increasing *N *and the low frequency resolution of the analysis, may allow parts of the system to demonstrate atypical or uninteresting behaviour. The potential consequence is that the quantitative significance of the interesting phenomena are reduced in the resulting frequency spectra, reducing the sensitivity of the technique. For the results presented here, values of *N *= 4000 and *δt *= 1 minute were chosen, corresponding to a frequency resolution of 0.00025 cycles per minute and a maximum observable frequency of 0.5 cycles per minute.

The result of the DFT is *N *complex numbers per simulation run, containing real and imaginary parts (equivalently, amplitude [Equation 2] and phase [$\arctan\left(f_{\omega }^{ℑ}∕f_{\omega }^{ℜ}\right)$]) for each of the *N *frequencies. Since these frequencies correspond exactly between runs (by virtue of the sampling), the data can be combined to give an average distribution. Note, however, that it is not sufficient to simply find the mean of the complex spectra. Since the DFT is a linear transformation, averaging the Fourier-transformed time series is equivalent to performing a Fourier transformation on the average of the time series. The result would suffer the same loss of behavioural information described above and illustrated in Additional file 1 Figure S7A. We overcome this problem by finding the mean of the *amplitudes *of the spectral data, where the amplitudes are given by:

(2) $${\hat{f}}_{\omega }=\sqrt{{\left(f_{\omega }^{ℜ}\right)}^{2}+{\left(f_{\omega }^{ℑ}\right)}^{2}}$$

${\hat{f}}_{\omega }$ is the *ω*^th ^component of the amplitude spectrum, $f_{\omega }^{ℜ}$ and $f_{\omega }^{ℑ}$are the real and imaginary parts of *f_ω_*, the *ω*^th ^component of the complex spectrum resulting from Equation (1). The average amplitude spectrum is then defined:

(3) $${\tilde{f}}_{\omega }=\frac{1}{K}\overset{K}{\underset{i}{\sum }}{\hat{f}}_{\omega ,i}$$

*K *is the number of simulation runs, ${\tilde{f}}_{\omega }$ is the *ω*^th ^component of the average amplitude spectrum, ${\hat{f}}_{\omega ,i}$ is the *ω*^th ^component of the amplitude spectrum from the *i*^th ^simulation run. By thus discarding the average phase information (noting that amplitude and phase are not independent in models of this kind and that phase information encapsulating the causality of individual traces is thus contained in the individual amplitude spectra), it is possible to reveal the average oscillatory behaviour in an intuitive way. We have found the average phase information to be less informative (highly stochastic, with no apparent coherence), although it can be examined independently, if required.

The spectra created in this way form distributions which tend to characterise the observed behaviour in a compact, informative form. Although the frequency spectra contain as many points as a single simulation run and may also contain noise, the processes of transformation and averaging serve to resolve and elucidate the characteristic behaviour. Moreover, we are then able to measure and compare the spectra so produced. In particular, we use a discrete space version of the Kolmogorov-Smirnov (K-S) statistic [55] as a measure of similarity between distributions:

(4) $$D=\max\left(\left|F_{N}^{1}-F_{N}^{2}\right|\right)$$

$F_{N}^{1}$ and $F_{N}^{2}$ are cumulative probability distributions of two frequency amplitude spectra ($\tilde{f}$ from Equation (3)) containing *N *elements. *D *is then a value in the interval [0, 1], where 0 corresponds to identical distributions. Our choice of this measure is based on the facts that its convergence characteristics are well understood, it has good discriminatory power and its calculation is efficient. The K-S statistic (resulting from a K-S *test*) is usually implemented in mathematical software as a function which takes the amplitude spectra directly as arguments. Note that to quantify the influence one species has on another it might be more appropriate to use information-theoretic measures such as *mutual information *or *cross entropy*.

The following procedure is used to generate average frequency spectra to characterise a set of simulations for the purpose of visual comparison or analysis of stochasticity.

Procedure A:

1. Perform a number of simulation runs which are long enough to demonstrate a phenomenon of interest.

2. Generate average frequency amplitude spectra for each molecular species:

a. Sample each simulation trace according to *N *and *δt*, chosen to suit the interesting phenomenon, and calculate a frequency amplitude spectrum based on Equations (1) and (2) using an FFT algorithm.

b. Calculate term-wise means of the amplitude spectra according to Equation (3).

3. Iterate 1 and 2, adding new simulations to the average as necessary (e.g., until the average spectra are sufficiently free of noise).

The following procedure is used to measure the difference between alternative systems or alternative simulation algorithms.

Procedure B:

1. Perform a number of pairs of simulation runs, where

a. each pair comprises the two alternative systems/algorithms and

b. the number of runs is designed to take an acceptable amount of time.

2. Generate average frequency amplitude spectra for each molecular species of the alternative systems/algorithms, as per Procedure A 2a and 2b.

3. For each molecular species of interest, calculate *D *according to Equation (4) applied to its average amplitude spectra from the alternative systems/algorithms, using a K-S test.

4. Iterate 1-3, adding new simulations to calculate *D*, until all *D*s are known with sufficient precision.

The number of simulation runs required (*K *in Equation (3)) is dependent on the inherent stochasticity of the systems under consideration and the resolution required. Insufficient simulation runs produce average distributions whose noise may obscure subtle differences in *D*. Informally, the number of simulations may be considered sufficient when the average spectra look smooth or when adding further simulations does not alter the order of the calculated values of *D *above some desired resolution threshold. In the present investigation, 100 runs reliably resolved differences in *D *of 0.05. For models which have a prohibitive computational cost of simulation it may be desirable to formalise the criteria for additional simulation runs to avoid unnecessary computation. One criterion might be to set a minimum acceptable coefficient of variation for spectral component means (${\tilde{f}}_{\omega }$). Alternatively, a sequential hypothesis test [56] could be used as the stopping criterion in Procedure B. The idea would be to set the supposed pair-wise order of various *D*s as null hypotheses and define desired probabilities of falsely rejecting a null hypothesis or falsely accepting the alternative. Each iteration would either confirm or reject the hypotheses, until the *stopping rule *indicates that the result is known with sufficient confidence. See [56] for details.

### Efficiency

Our analysis methodology scales efficiently with respect to model size (number of different molecular species), especially in comparison to numerical techniques for finding the probability distribution of states in Markov chains (the mathematical structure underlying our stochastic models) [57]. Since the state space scales exponentially with model size, such techniques rapidly become intractable. Moreover, expressing properties of behaviour in terms of frequency using these techniques is cumbersome at best. Statistical approaches based on the same measures, but which circumvent enumerating the state space by using simulation, suffer the same limitation when expressing frequency. The principal computational cost of our technique is the simulation runs: the DFT is performed by a standard Fast Fourier Transform (FFT) algorithm on only a small subset of points from each simulation trace. The size of this subset is essentially independent of the number of molecules and reactions in the system and is only related to the bandwidth of the phenomena being compared. The length of a simulation run is the total number of reaction events that take place. In a system comprising isolated subsystems, the total number of reaction events is the sum of the reaction events in each subsystem - linear scaling. In a system where the subsystems are coupled, additional reaction events take place when the subsystems interact. If such reactions apply to just a few coupling species and the behaviour of the subsystems does not change radically (as in the present investigation), the overall effect of coupling on efficiency is minimal. Under other circumstances, the increase in computational cost still only scales with a low order polynomial.

## Results and discussion

**Figure 1:**
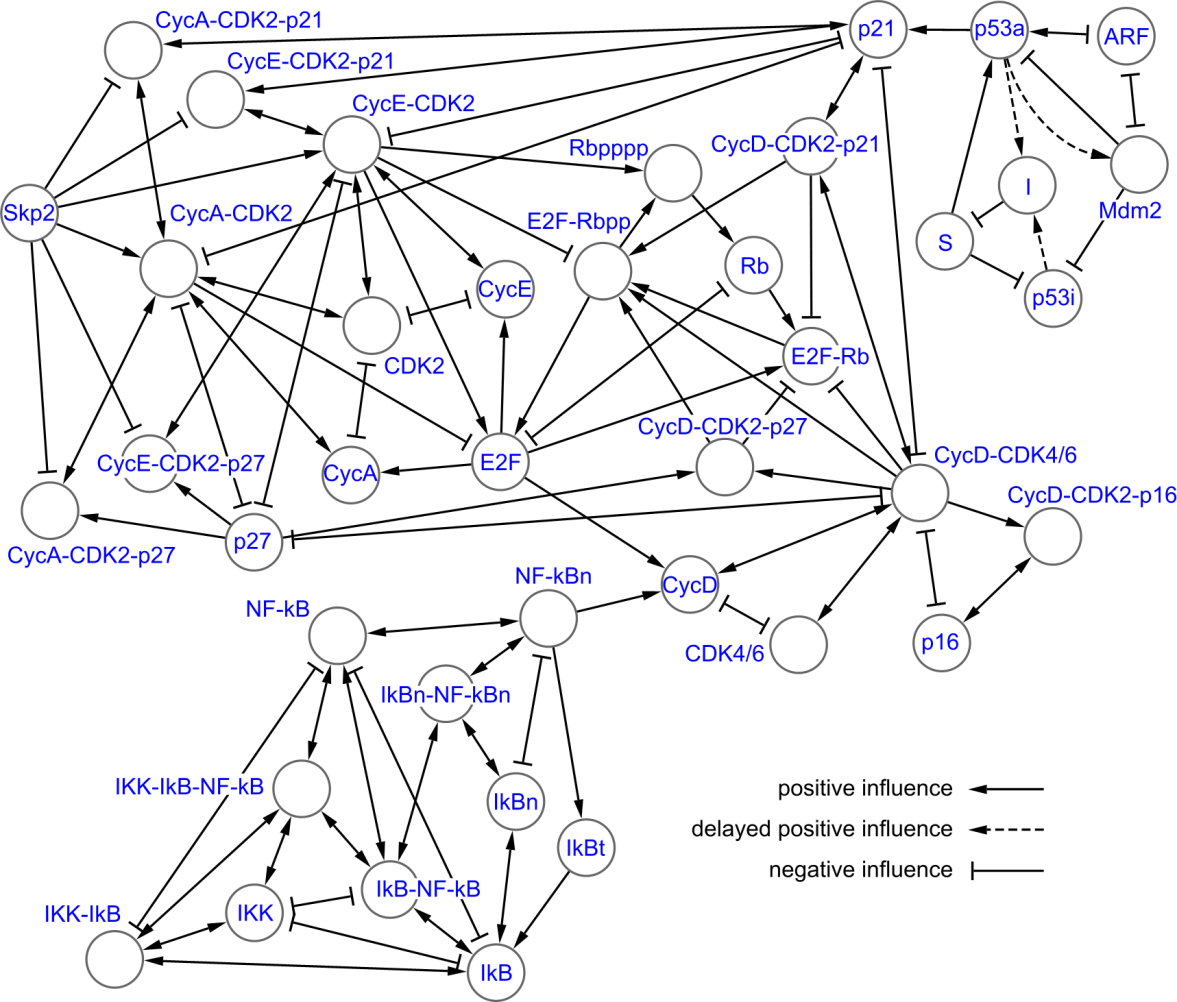
**Coupled model of cell cycle G1/S phase, p53 and NF-κB**. Diagram showing the complete model described in the text, illustrating how molecular species influence those to which they are connected by reactions. A complete mathematical description of the model is given in Additional file 1.

Our crosstalk experiment considers the vector of change comprising the changes in behaviour of molecular species in the cell cycle resulting from connection to the NF-κB and p53 systems, relative to their behaviour when the external systems are not connected. Precise details of the models we constructed are given in Additional file 1, while Figure 1 contains a diagrammatic representation of the fully coupled system.

**Figure 2:**
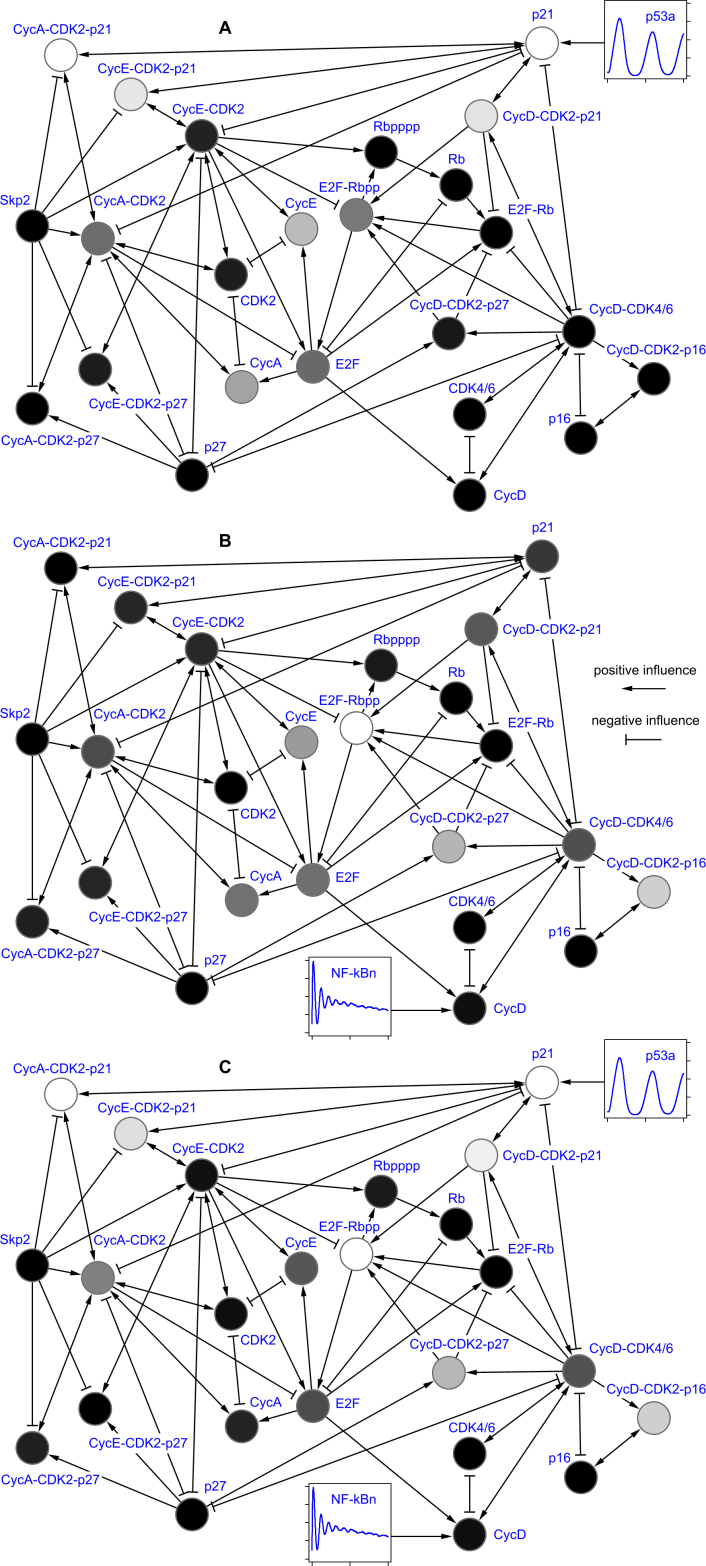
**Perturbation of cell cycle components by p53a and NF-κBn**. Diagrams illustrate the quantitative influence of external oscillatory networks (not depicted) on cell cycle components (the nodes). White nodes are most perturbed, black nodes least (values in Additional file 1 Table S1). Arrows denote direction and nature of influence. **A **Perturbation by p53a. **B **Perturbation by NF-κBn. **C **Perturbation by simultaneous influence of p53a and NF-κBn.

We applied Procedure B (Methods) with pairs of *K *= 100 simulation runs to calculate values of *D *(Equation (4)) for every species in the cell cycle model; for the cell cycle coupled to the p53 pathway alone, the cell cycle coupled to the NF-κB pathway alone and the cell cycle coupled to both. The numerical values are tabulated in Additional file 1 Table S1 and illustrated in Figure 2. In what follows we use the term perturbation to mean these values and equivalently refer to perturbation by p53a and NF-κBn, since p53a and NF-κBn are the molecular species which link the respective systems to the cell cycle in our model. In order to validate our choice of parameters for the coupling reactions we also investigated the effects of double and ten times increased coupling strength. The results of this are tabulated in Additional file 1 Tables S2 and S3 and illustrated in Additional file 1 Figures S1 - S6.

Figure 2A illustrates the effect on the cell cycle by p53a alone. We see that p21 is strongly perturbed, as we might expect given that it is directly influenced by p53a, and that some of the species directly influenced by p21 are also moderately or strongly perturbed. Less intuitively, we see that some species directly influenced by p21 are only weakly perturbed (CycE-CDK2, CycD-CDK4/6), while other species indirectly connected are strongly perturbed. The effect of NF-κBn (Figure 2B) is even less intuitive. The species directly influenced by NF-κBn (CycD) is only weakly perturbed, while species as far as five steps away in the network (i.e., CycA, CycE) are moderately perturbed. With simultaneous influence by both p53a and NF-κBn (Figure 2C) the pattern of perturbation broadly follows that expected by combining the individual cases. Note, however, that CycA and CycE (moderately perturbed at a distance of five steps from NF-κBn) are *less *perturbed in the full system. The level of perturbation is apparently dependent on how the perturbed species 'resonates' with the perturbation and is therefore both amplitude and frequency dependent. Moreover, we see that the magnitude of perturbation is not in general cumulative.

Previous work [52] has shown that the technique of frequency domain analysis applied here is especially revealing when applied to stochastic simulation traces; the variance found in reality being absent in deterministic simulations. While most of our results therefore concern stochastic simulations, we duplicated many of our experiments using quasi-deterministic models and used the presented technique of frequency domain analysis to investigate the differences. The quasi-deterministic models are constructed from exactly the same reaction scheme and kinetic parameters, however the kinetic functions for production and consumption of a particular species are combined in a single, resultant, function, as in the case of deterministic simulations (see Additional file 1 supplementary methods). This function is then simulated stochastically (hence *quasi*-deterministic), but produces much less stochasticity than the reaction-based model. By maintaining the same discrete state space and simulation framework between the two types of models, it is possible to resolve the effects of stochasticity more clearly and avoid the artefacts sometimes created by deterministic solvers. Moreover, we are able to visualise and quantify the often cited 'inaccuracy' of not converting systems to elemental reactions. The artefacts of deterministic solvers and the relationship between deterministic, quasi-deterministic and stochastic simulations are illustrated in Additional file 1 Figure S7B.

**Figure 3:**
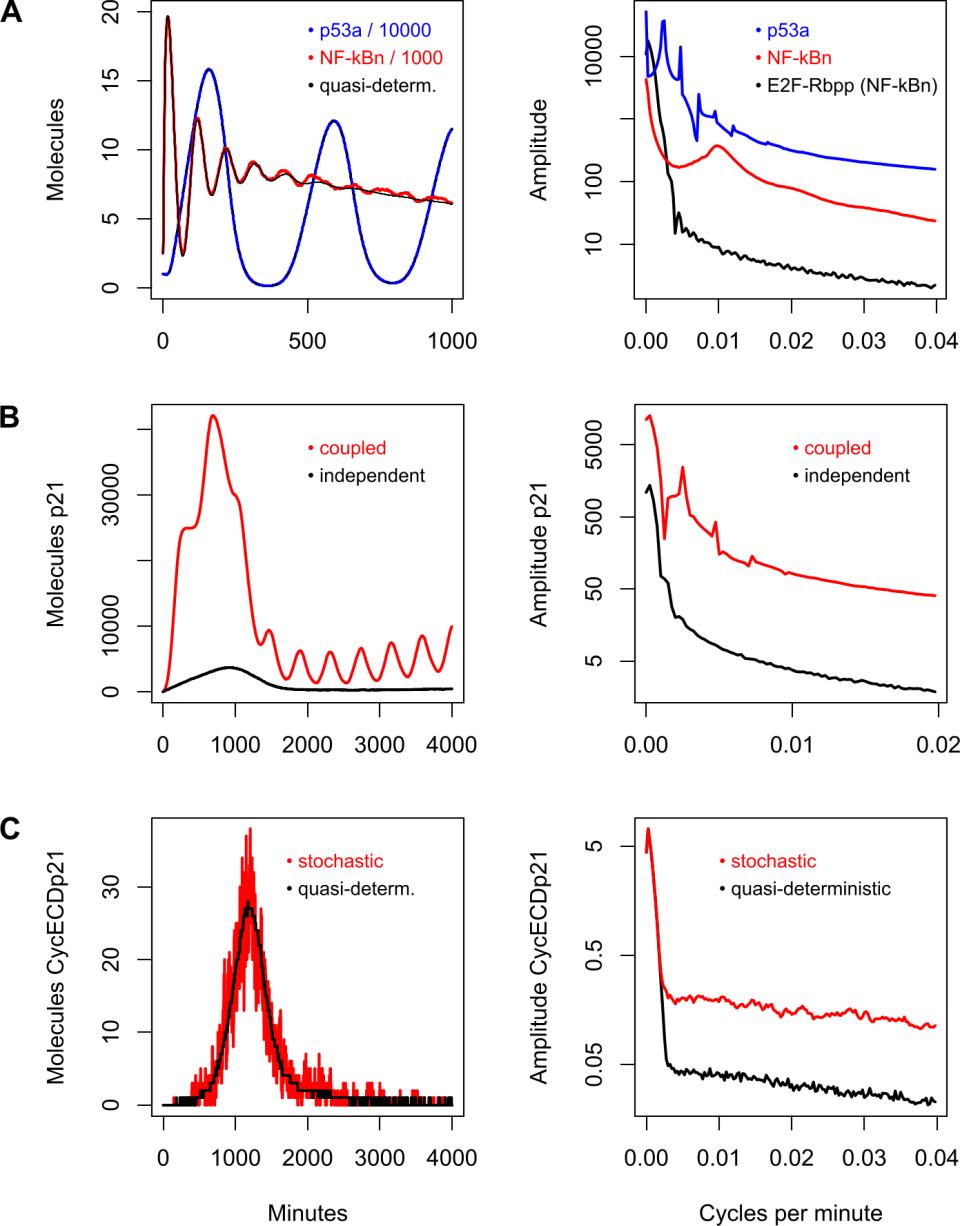
**Time and frequency domain representations of the behaviour of NF-κBn, p53a, p21 and CycE-CDK2-p21**. Individual time courses (left) and average frequency spectra (right). **A **Left panel: time courses of stochastically simulated NF-κBn (red) and p53a (blue). Quasi-deterministic time courses superimposed in black. Right panel: average frequency spectra of NF-κBn (red), p53a (blue) and E2F-Rbpp perturbed by NF-κBn alone (black). **B **Evidence of crosstalk in time (left) and frequency domain (right) of p21 in the fully coupled network (red), in comparison to the isolated cell cycle (black). **C **Stochasticity in time (left) and frequency domain (right) of CycE-CDK2-p21 in isolated cell cycle, using quasi-deterministic (black) and fully stochastic models (red).

Figure 3A shows time and frequency domain representations of p53a and NF-κBn in the fully stochastic model. For comparison, the time series of NF-κBn in the quasi-deterministic model is also shown in the left hand panel (black). Note the order of magnitude difference in scales for the two molecules. p53a has greater amplitude and a primary oscillatory mode approximately five times slower than that of NF-κBn. While stochasticity is minimal in these species, the stochastic model of NF-κBn has qualitatively different behaviour to the quasi-deterministic model from about 500 minutes onwards; whereas oscillations die out in the quasi-deterministic model, they apparently continue in the stochastic model. We speculate that this is due to the system being neutrally stable at this point and the stochastic noise 're-ignites' the oscillatory behaviour. The corresponding frequency spectra (Figure 3A, right panel) are shown on a logarithmic scale to reveal more detail at higher frequencies. Note that the primary oscillatory mode of p53a, at around 0.002 cycles per minute, is similar in magnitude to the peak corresponding to its initial transient near zero. By contrast, the principal oscillatory mode of NF-κBn, at about 0.01 cycles per minute, is an order of magnitude lower than the peak corresponding to its transient. This perhaps explains why we find that the oscillatory mode of NF-κBn is less apparent in those species of the cell cycle that it perturbs, with or without the presence of the p53 system. This is illustrated in the right panel of Figure 3A, which shows the average spectrum of E2F-Rbpp when strongly perturbed by NF-κBn alone: there is no evidence of the characteristic oscillatory signature of NF-κBn.

Figure 3B shows the effect of p53a on p21 in the fully coupled model in the time and frequency domains. In the time domain (left panel), the initial transient peak at around 1000 minutes in the isolated cell cycle is amplified considerably in the coupled model and the oscillatory frequency of p53a is clearly visible. In the frequency domain, the initial transient is represented by the peak near zero (which extends beyond the axes in the case of the coupled model), while the characteristic harmonic peaks of the oscillation of p53a appear higher up in the frequency spectrum of p21.

Figure 3C shows the effect of stochasticity on CycE-CDK2-p21 in the quasi-deterministic and fully stochastic models. In the time domain there is no apparent structure to the noise evident on the red curve (fully stochastic), noting that the black curve (quasi-deterministic) has much less noise. In the frequency domain, the noise manifests itself as an apparently constant value added to the spectrum of the quasi-deterministic spectrum above about 0.003 cycles per minute. Due in part to the generally large numbers of molecules in the three sub-networks that comprise the system, stochasticity does not appear to play a significant role in our findings. Note in particular from Additional file 1 Table S4 that the two species that directly perturb the cell cycle (namely p53a and NF-κBn) have low stochasticity, so any noise inherent in their respective networks does not propagate to the cell cycle. Some molecular species of the cell cycle, such as CycE-CDK2-p21, do indeed remain in low copy number and show significant stochasticity; however, due to the structure (e.g. negative feedback) and parameters of the network, their influence on overall behaviour is minimal. The effects of stochasticity on individual species are tabulated in Additional file 1 Table S4.

**Figure 4:**
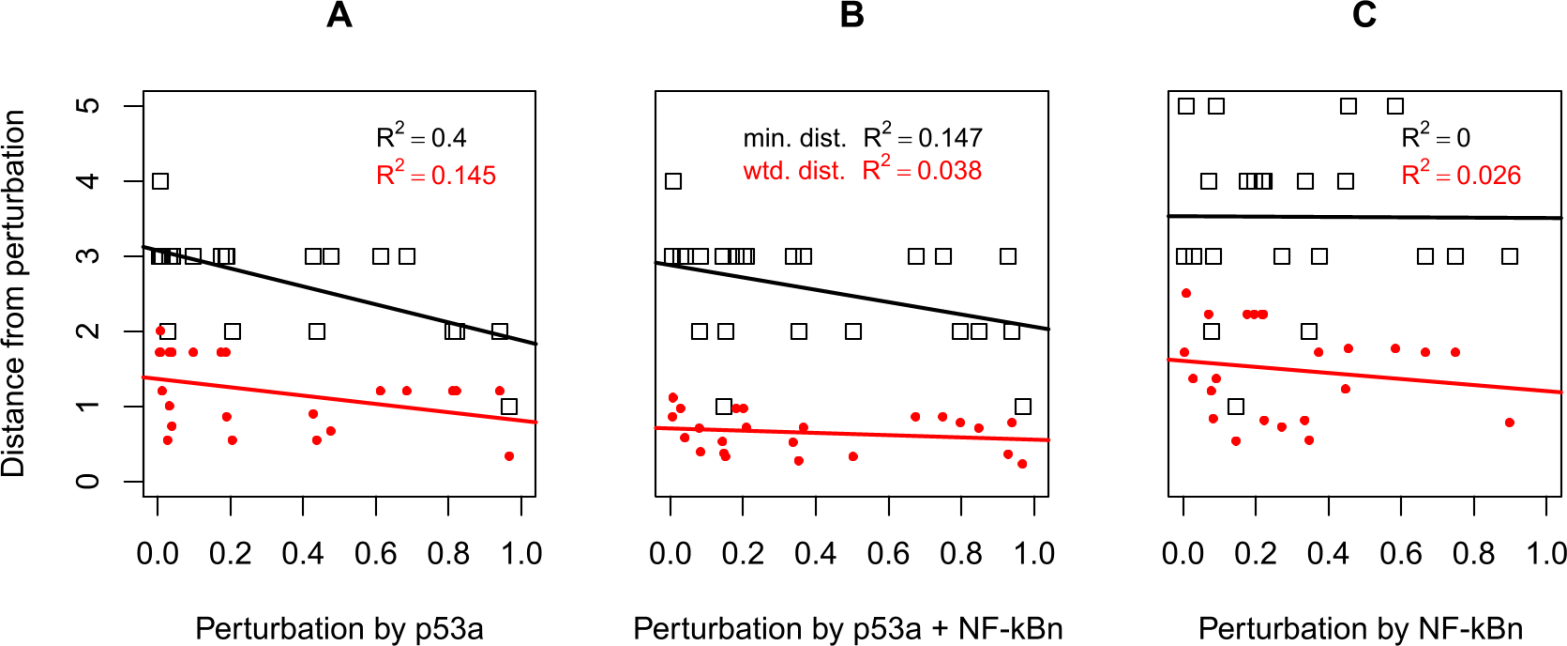
**Evaluation of network-based heuristics by frequency domain analysis**. Correlation of minimum distance (black) and weighted network distance (red) with measured perturbation of the cell cycle. **A **Perturbation by p53a. **B **Simultaneous perturbation by p53a and NF-κBn. **C **Perturbation by NF-κBn. R^2 ^is the coefficient of determination and indicates the ability of the heuristic to predict the measurement: R^2 ^= 1 is perfect; R^2 ^= 0 shows no ability.

It is immediately apparent from our results that the nature of crosstalk is at times counter-intuitive in terms of causality. For example, the species directly influenced by NF-κBn is only weakly perturbed while the point of maximum perturbation is three steps away from NF-κBn. Such phenomena are perhaps to be expected in coupled non-linear dynamical systems. Nevertheless, we wished to investigate whether there is in fact a simpler explanation of crosstalk, based on network topology, that can be inferred without simulation. In Figures 1 and 2 the nodes are linked by lines indicating the direction and nature (positive or negative) of the influence one species has on another. In general, species A has a positive influence on species B when A is a substrate or enzyme for the production of species B: the more A that exists, the more B is produced. Species A has a negative influence on species B when B is consumed in a reaction and A is either an enzyme or substrate in the same reaction: the more A that exists, the more B is consumed. Using this network abstraction we evaluated the correlation between the distance from the source of influence to each species in the network and the crosstalk measured using our frequency domain analysis technique. Figure 4 charts the results considering the minimum distance (the minimum number of steps in the network) and the weighted distance (combining the effects of all possible paths, weighted inversely proportional to their length). Figure 4A shows that the relatively simple measure of minimum distance (black) is apparently able to adequately characterise the measured perturbation caused by connecting the cell cycle to the p53 system. By contrast, Figure 4C demonstrates that the minimum distance is a completely inadequate model of the perturbation caused by the NF-κB system; the corresponding coefficient of determination (R^2^) value of 0 indicates that the minimum distance has no predictive power in this case (R^2 ^= 1 being perfect). By including the influence of all possible paths between NF-κBn and cell cycle species the predictive power of the model improves (red). In the case of influence by p53a (Figure 3A), however, considering all paths actually reduces the predictive power of the model (R^2 ^= 0.145 vs. R^2 ^= 0.4). In the fully coupled model (Figure 4B) we observe a similar diminution; considering all paths has only weak predictive power.

Thus the prediction afforded by the minimum distance may at times *appear *to be good but at other times is completely erroneous, while the weighted sum of paths gives an overall weak performance. These results clearly indicate the dangers of using heuristics without validation by a reliable benchmark. We also considered (but do not show) weighted distances incorporating the nature (positive or negative) of interactions, such that the length of any path is taken to be either positive or negative depending on the cumulative nature of the individual steps along it. Despite this additional information, however, we found that this was less satisfactory than when we excluded such phase considerations. Since the rates of reactions and the concentrations of species may effectively (and dynamically) alter the topology of the network, it is not surprising that it is difficult to encapsulate the subtle non-linear frequency-dependent interplay when these are excluded.

## Conclusions

A key challenge of systems biology is to assemble the disparate information gathered over years of experimentation and research into a coherent whole. To avoid the intractable computational cost of re-parameterising existing models, heuristic techniques, such as those of network analysis, may be employed to simplify the task. To evaluate the performance of these heuristics and verify what is created, efficient, meaningful, high resolution analytic techniques must be developed. This document presents one such: a systematic technique for characterising behaviour and for measuring the interactions and connections between and within signal transduction pathways using frequency domain analysis. We have constructed a novel dynamical model of communicating oscillatory networks of p53, NF-κB and the G1/S phase of the cell cycle and have applied our technique to investigate it. In doing so, our investigation has revealed complex counter-intuitive dependencies and has demonstrated that the methodology is reliable, precise and capable of distinguishing the effects of multiple interactions.

As general conclusions for the model we have found that (i) p21 and CycA-CDK2-p21 are the species most strongly influenced by the p53 network and that the perturbation is primarily at the principal oscillatory frequency of p53a and local to the perturbation; (ii) p21 and CycA-CDK2-p21 are only weakly perturbed by the NF-κB network; (iii) E2F-Rbpp is the species most strongly perturbed by the NF-κB network and the perturbation is indirect and from the low frequency transient of NF-κBn, rather than its higher frequency oscillations; (iv) increased coupling strength tends to reinforce trends in crosstalk; however (v) E2F-Rbpp is moderately perturbed by p53a with single coupling strength, *less *perturbed with double strength coupling and again moderately perturbed with ten times coupling strength; (vi) species E2F-Rb, p16, Rb and Skp2 remain unperturbed for all combinations of perturbations and coupling strengths. In the case of Skp2 this can be immediately inferred from the topology; it is not influenced by other species. We might also expect p16 to be only weakly perturbed because it has both positive and negative influence derived from a single species (CycD-CDK4/6). Positive and negative influence do not in general cancel each other (especially when the influence is at different frequencies) and we have shown that network topology alone is an unreliable indicator of influence.

Quantifying in detail the extent to which molecular species are robust or sensitive to perturbations potentially indicates the mechanisms by which the system may be manipulated in experiments and therapeutics. Strictly, the dependencies we have discovered are features of the models we have used, the simulation algorithm we have chosen and the links we have hypothesised (the standard modeller's proviso). There are clearly many additional interconnections with other pathways that we (and others) have not yet modelled (the published models of the systems we consider here are continually being refined [58-66]), but given that the individual models with which we started are experimentally validated and of high quality, that we guarantee our conversion procedure maintains their original properties while making them more closely respect the underlying physical processes and that our simulation algorithm is rigorous, it is reasonable to assume that our results say something about the real biological systems.

We have described how our methodology is efficient with respect to the standard numerical techniques used to investigate Markov chains and have observed that, in addition, such techniques are cumbersome in describing behaviour in comparison to ours. To add weight to these claims and as a further demonstration of the utility of our benchmark technique, we have shown the results of investigating two network-based heuristics, finding that they are not adequate in describing the complex frequency-dependent interplay in our model and may give misleading results. It is important to note here that our methodology is a precise means of measuring and comparing simulation time series and that it has no obvious inherent prejudice with respect to the type of model or means of simulation. There are practical considerations, relating to the efficacy and precision of numerical algorithms, which make certain combinations of model and simulation algorithm infeasible, but these considerations are independent of our methodology. In our investigation of the cell cycle - p53 - NF-κB system, we have used an exact stochastic simulation algorithm, but have chosen to investigate both a model which is, as far as possible, reduced to elemental reactions (thus modelling the supposed real physical process) and one which is essentially a stochastic interpretation of the differential equations (perhaps only weakly related to physics). While the qualitative differences between these two cases is clear, our methodology is able to provide a *quantification *of the differences and, importantly, can do so when the differences are not known a priori.

Our focus has been stochastic models, but there are well-established techniques used to investigate the dynamics of deterministic systems that can be seen as potential alternatives to our methods (ignoring their fundamental limitation of not considering variance). *Algebraic analysis *tends to become infeasible for dynamical systems of greater than five dimensions (unless there is significant symmetry or possible simplification), hence the principal deterministic analytic technique is simply to numerically solve the set of differential equations that describe the system, by simulating a trajectory in time from some initial state. *Phase plane analysis *can reveal the qualitative features in the state space (stable and unstable fixed points etc.) which account for the dynamics of systems with two dimensions or which can be reasonably simplified to two dimensions. This represents a very small class of systems and such techniques do not scale. *Bifurcation analysis *of a system may be used to find the critical dependence of its equilibria and fixed points (which define its dynamics) on parameters, however this does not necessarily quantify or characterise the typical behaviour of the system. *Sensitivity analysis *is often used with deterministic models to identify their most important parameters by quantifying the changes of behaviour (according to some statistical model) with respect to changes of the parameters. Such an approach is not limited to deterministic systems and would, we suggest, be more effective using our frequency domain-based definition of behaviour. Overall, existing techniques used on deterministic systems tend to be somewhat ad hoc, depending on the intuition of the investigator, and do not allow the convenient quantification of behaviour that our methodology provides.

Given the vast repository of individual models in the literature and in online databases that await combination and validation, we have shown that our methods have great potential for application in systems biology. We also envisage further improvements and refinements to our techniques. Biological systems often contain processes working at orders of magnitude different scales of time and size. Although transformation into the frequency domain has here proved to be both effective and intuitive, in order to integrate and analyse large *multi-scale *systems, we feel it may be efficacious to consider the more abstract *wavelet *transformation.

## Competing interests

The authors declare that they have no competing interests.

## Authors' contributions

AECI created the models and co-wrote the manuscript. SAS performed the simulations and analysis and co-wrote the manuscript. The authors read and approved the final manuscript

## Supplementary Material

Additional file 1 **Supplementary material**. The supplementary material contains supplementary results and methods, including details of the mathematical models employed and other examples of the application of frequency domain analysis. Click here for file
