# Supplementary material for: Communicating oscillatory networks: frequency domain analysis
Source: BMC Syst Biol. 2011 Dec 22;5:203. doi: 10.1186/1752-0509-5-203 (PMC3287135; doi:10.1186/1752-0509-5-203)
Supplement: Additional file 1 — Supplementary material. The supplementary material contains supplementary results and methods, including details of the mathematical models employed and other examples of the application of frequency domain analysis. [file 1752-0509-5-203-S1.pdf]

Additional file 1 for
'Communicating oscillatory networks: Frequency Domain Analysis'

Table of contents

Supplementary results ... 2
Crosstalk between oscillatory models of the cell cycle G1/S phase, NF-κB and p53 ... 2
Table S1: Crosstalk in the cell cycle coupled to p53, to NF-κB and both p53 and NF-κB. ... 2
Increased coupling strength ... 2
Figure S1: Perturbation of the cell cycle by p53a with double coupling strength. ... 3
Figure S2: Perturbation of the cell cycle by NF-κBn with double coupling strength. ... 4
Figure S3: Perturbation of the cell cycle in full model with double coupling strength. ... 4
Figure S4: Perturbation of the cell cycle by p53a with ten times coupling strength. ... 5
Figure S5: Perturbation of the cell cycle by NF-κBn with ten times coupling strength. ... 5
Figure S6: Perturbation of the cell cycle in full model with ten times coupling strength. ... 6
Table S2: Crosstalk in the cell cycle coupled to p53, to NF-κB and both p53 and NF-κB with double coupling strength. ... 7
Table S3: Crosstalk in the cell cycle coupled to p53, to NF-κB and both p53 and NF-κB with ten times coupling strength. ... 8
The effect of stochasticity on independent networks ... 9
Table S4: Differences between reaction-based and quasi-deterministic models in the absence of coupling. ... 9
Supplementary methods ... 10
NF-κB system ... 10
Cell cycle system ... 10
DNA damage transduction system ... 11
Stochastic delay differential equations ... 12
Model naming convention ... 13
Stochastic (reaction-based) models ... 14
p53 ... 14
Cell cycle ... 14
NF-κB ... 16
Coupling reactions ... 16
Quasi-deterministic (combined production / consumption) models ... 17
p53 ... 17
NF-κB ... 17
Cell cycle ... 18
Coupling reactions ... 18
Rate and other constants ... 18
Initial numbers of molecules ... 20
Supplementary example: a stochastic model of the eukaryotic cell cycle ... 20
Figure S7: Time and frequency domain behaviour of CycBT in generic eukaryotic cell cycle 22
Table S5: Stochastic model of the generic eukaryotic cell cycle ... 23
References ... 24

Supplementary results

Crosstalk between oscillatory models of the cell cycle G1/S phase, NF-κB and p53

Table S1 gives the numerical values used to generate Figures 2A-C in the main text.

NF-κB – cell cycle	*D*	p53 – cell cycle	*D*	p53 – NF-κB – cell cycle	*D*
E2F-Rbpp	0.899	p21	0.968	p21	0.969
CycD-CDK2-p16	0.75	CycA-CDK2-p21	0.941	CycA-CDK2-p21	0.94
CycD-CDK2-p27	0.667	CycE-CDK2-p21	0.822	E2F-Rbpp	0.93
CycE	0.585	CycD-CDK2-p21	0.812	CycD-CDK2-p21	0.849
CycA	0.455	CycE	0.685	CycE-CDK2-p21	0.798
E2F	0.447	CycA	0.613	CycD-CDK2-p16	0.75
CycD-CDK2-p21	0.374	E2F-Rbpp	0.476	CycD-CDK2-p27	0.675
CycD-CDK4/6	0.348	CycA-CDK2	0.438	CycA-CDK2	0.503
CycA-CDK2	0.335	E2F	0.428	CycE	0.366
p21	0.272	CycE-CDK2	0.205	CycD-CDK4/6	0.353
CycE-CDK2	0.224	CDK2	0.19	E2F	0.338
CycE-CDK2-p21	0.222	CycE-CDK2-p27	0.188	CycA	0.209
CycE-CDK2-p27	0.217	CycD-CDK2-p27	0.174	CycA-CDK2-p27	0.201
CycA-CDK2-p27	0.196	Rbpppp	0.098	Rbpppp	0.181
Rbpppp	0.177	CycA-CDK2-p27	0.04	CycE-CDK2	0.152
CycD	0.146	E2F-Rb	0.039	CycD	0.148
CDK2	0.092	p27	0.032	CDK2	0.144
E2F-Rb	0.083	CycD-CDK2-p16	0.032	E2F-Rb	0.083
CDK4/6	0.078	CycD-CDK4/6	0.027	CDK4/6	0.08
CycA-CDK2-p21	0.071	CycD	0.012	p27	0.04
p27	0.028	Skp2	0.011	CycE-CDK2-p27	0.028
Rb	0.009	CDK4/6	0.008	Skp2	0.008
Skp2	0.007	Rb	0.008	Rb	0.007
p16	0.004	p16	0.005	p16	0.006

Table S1: Crosstalk in the cell cycle coupled to p53, to NF-κB and both p53 and NF-κB. Each species of the cell cycle is assigned a *D* value (Equation (4)) in the interval [0,1] by Procedure B (Methods), where larger values indicate greater crosstalk. Values less than 0.1 correspond to amounts of crosstalk which are difficult to discern in the time and frequency plots. In each case, species are listed in descending order of *D*.

Increased coupling strength

The coupling strength of the oscillatory systems is controlled by the values of rate constants R30b and R40b. The initial coupling strength was chosen to be plausible and to respect known parameters and experimentally verified behaviour. To validate our results, we also considered twice and ten times this coupling strength. Figure S1 illustrates the perturbation (*D*) of cell cycle components by p53a using double coupling strength. While the trend of crosstalk is reinforced by most components, in line with intuition, it is noticeable that E2F-Rbpp is apparently influenced *less* with increased coupling strength (D = 0.178 vs. 0.476). With ten times coupling strength (Figure S4) this

unexpected trend is partially reversed (0.369). This counter-intuitive behaviour is to be expected in non-linear dynamical systems that are sensitive to both the amplitude and frequency of perturbations. Overall, Figures S1-S6, variously illustrating the influence of NF-κB alone, p53 alone and the fully coupled systems with double and ten times coupling strength, tend to follow a more or less intuitive trend of increased measured perturbation with increased coupling. Whereas with single coupling strength the influence of p53a tends to be local and the influence of NF-κBn tends to be indirect, with increased coupling strength more species are affected to a greater degree and this demarcation is blurred. There are some noticeable exceptions, however. Species E2F-Rb, p16, Rb and Skp2 are minimally perturbed by any combination of coupling and coupling strengths. Values for double and ten times coupling strength are given in Tables S2 and S3, respectively.

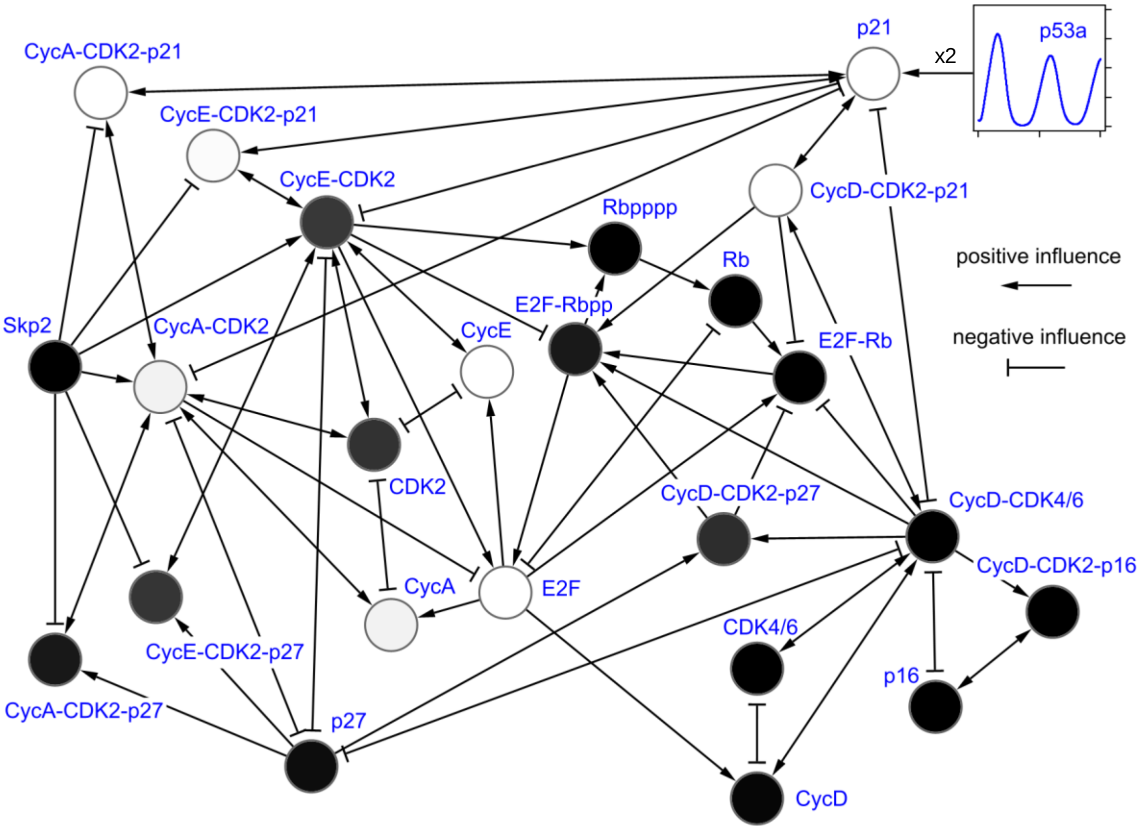

Figure S1: Perturbation of the cell cycle by p53a with double coupling strength. While the pattern of crosstalk evident in Figure 2 is broadly reinforced with increased coupling strength, contrary to intuition the perturbation of species E2F-Rbpp is reduced. Values of perturbation (*D*) are given in Table S2.

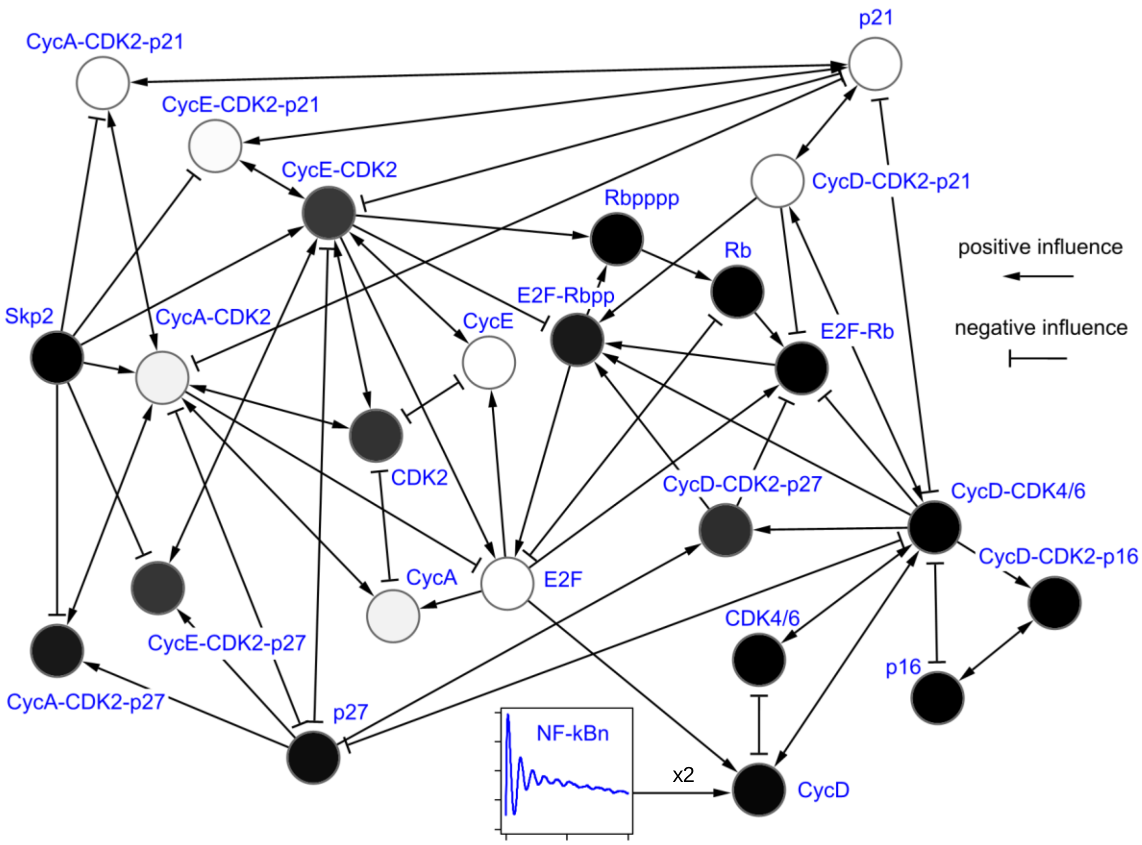

Figure S2: Perturbation of the cell cycle by NF-κBn with double coupling strength.
Values are given in Table S2.

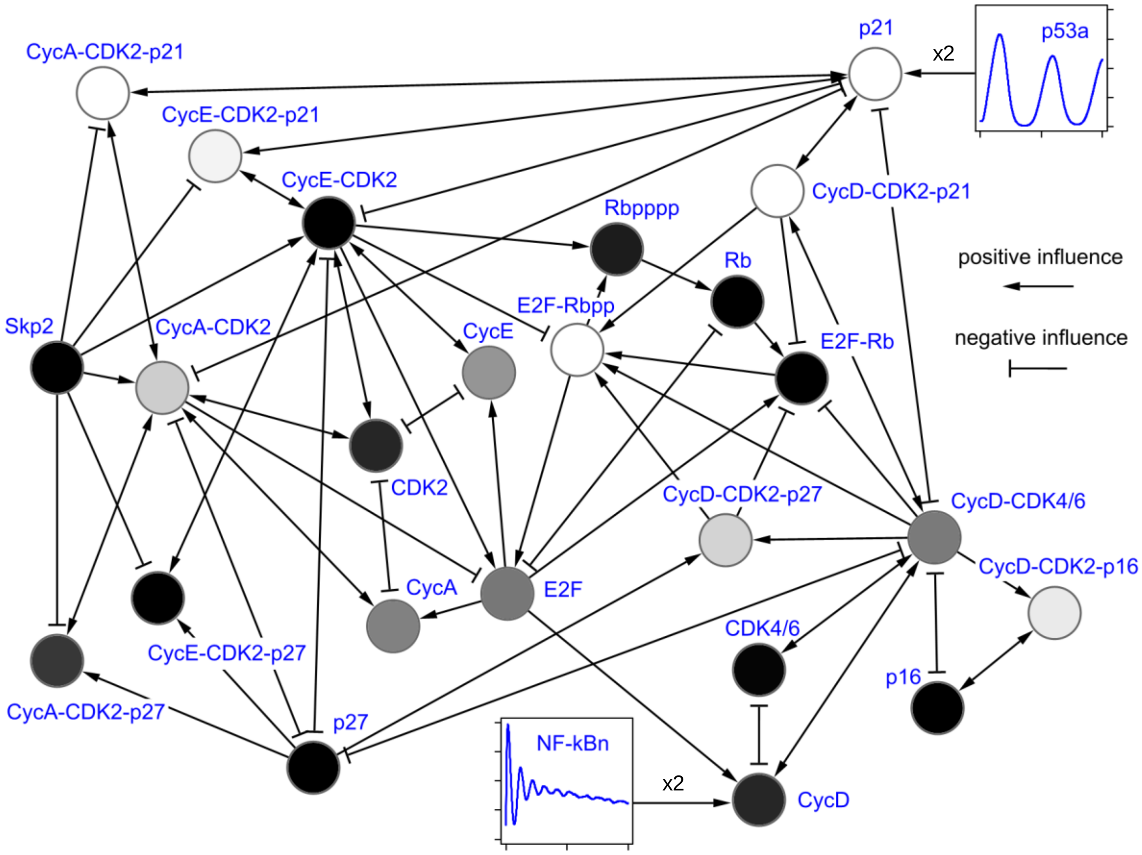

Figure S3: Perturbation of the cell cycle in full model with double coupling strength.
Values are given in Table S2.

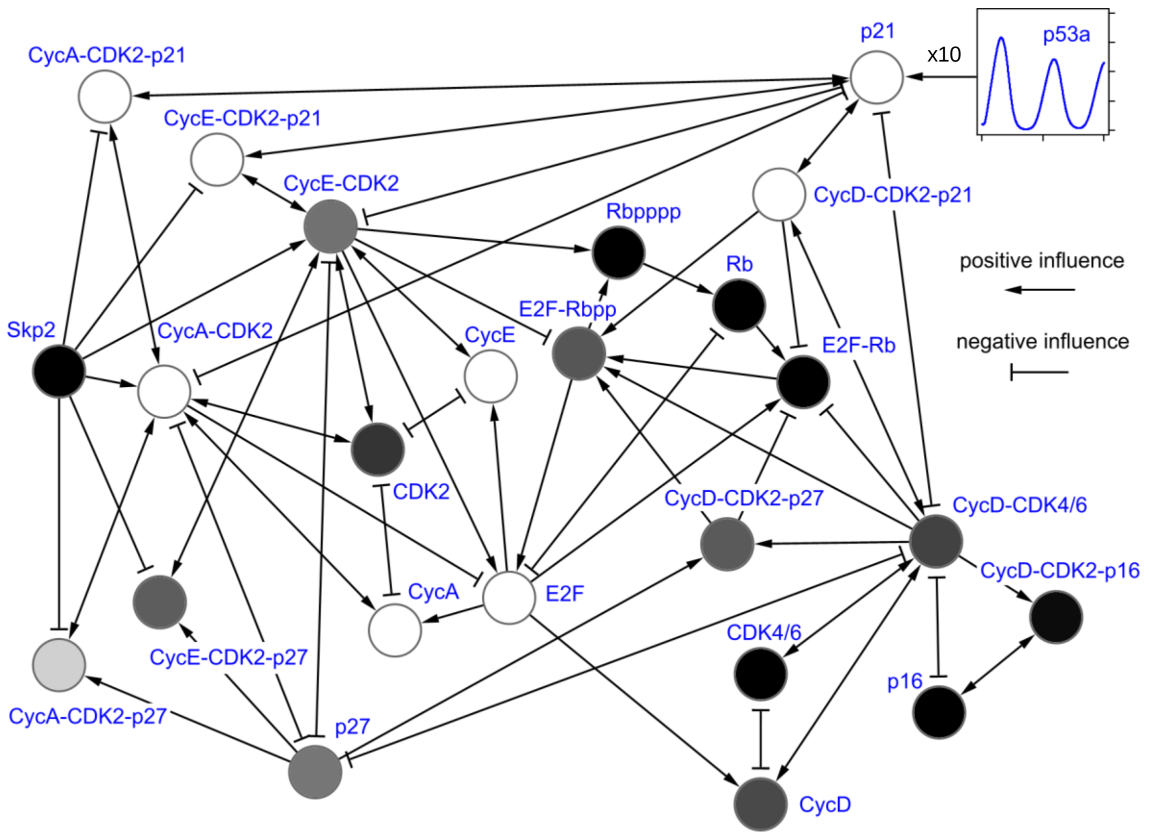

Figure S4: Perturbation of the cell cycle by p53a with ten times coupling strength. Values are given in Table S3.

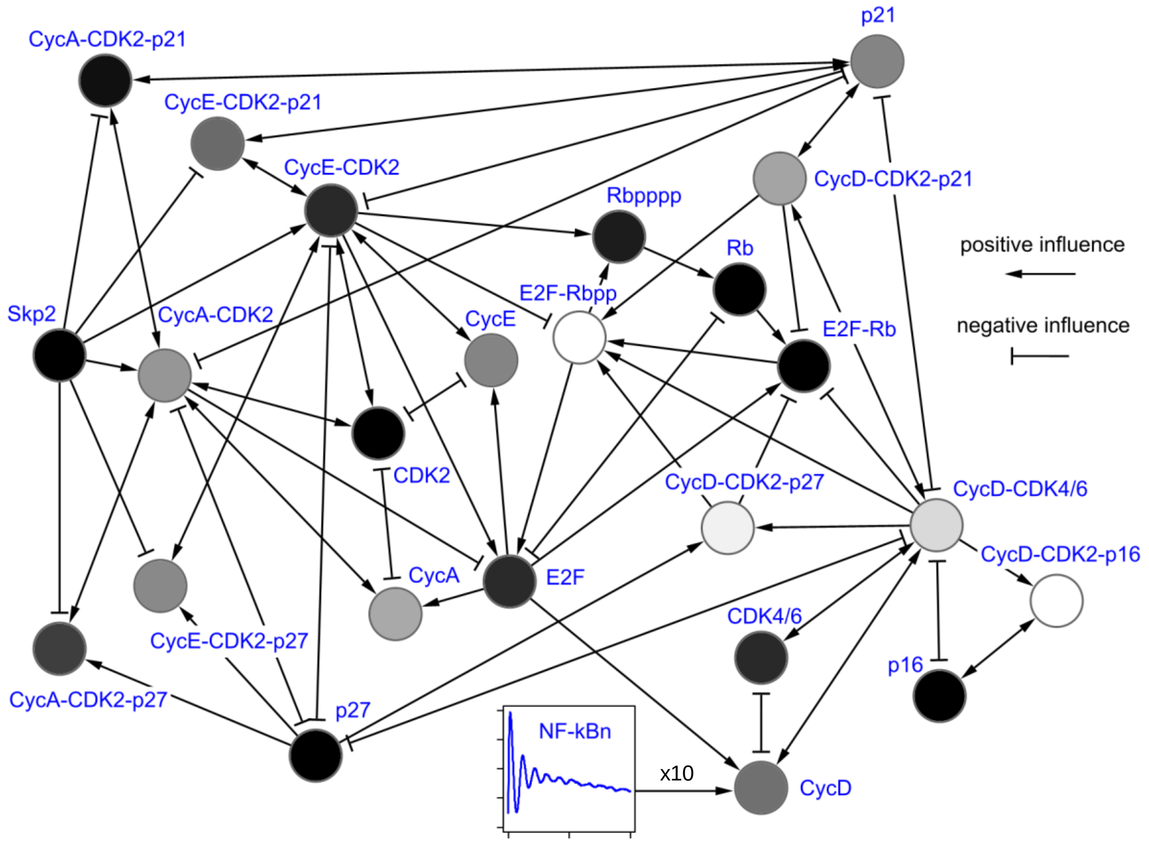

Figure S5: Perturbation of the cell cycle by NF-κBn with ten times coupling strength. Values are given in Table S3.

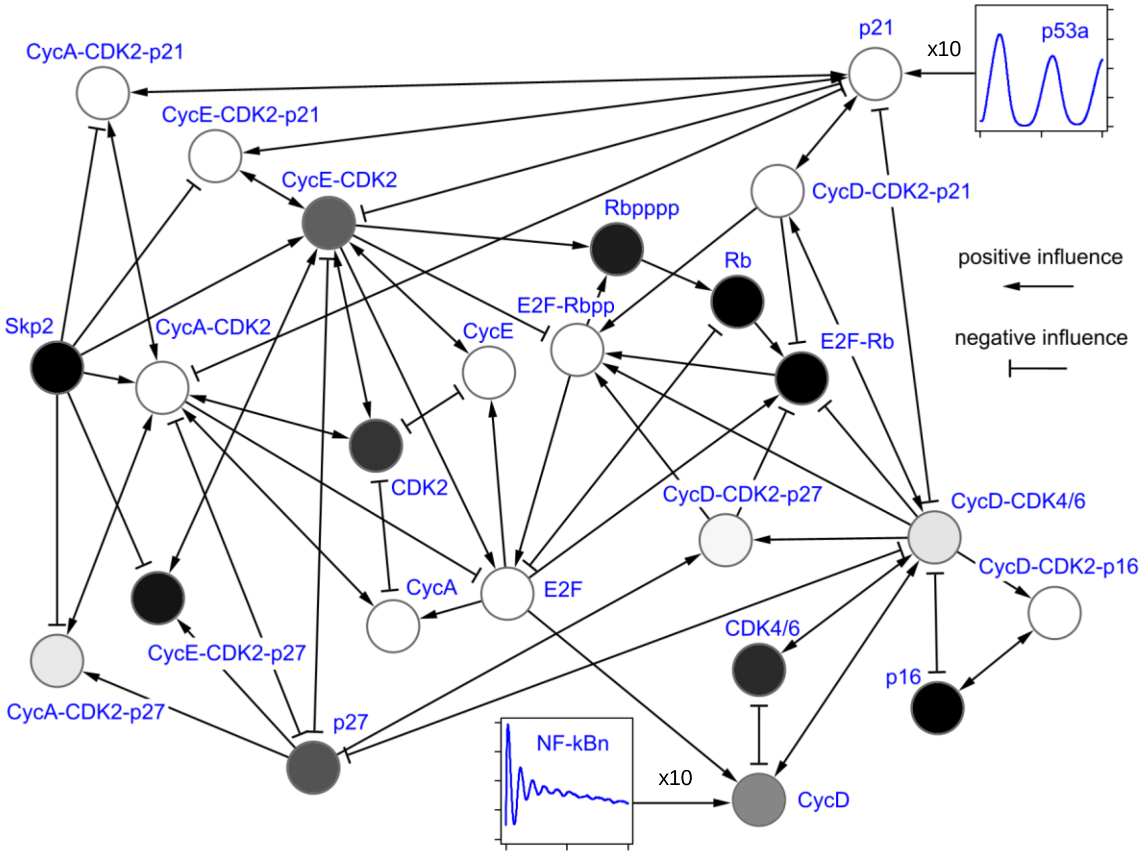

Figure S6: Perturbation of the cell cycle in full model with ten times coupling strength. Values are given in Table S3.

NF-κB – cell cycle	*D*	p53 – cell cycle	*D*	p53 – NF-κB – cell cycle	*D*
E2F-Rbpp	0.943	p21	0.993	p21	0.989
CycD-CDK2-p16	0.836	CycE	0.969	CycA-CDK2-p21	0.968
CycD-CDK2-p27	0.754	CycA-CDK2-p21	0.967	E2F-Rbpp	0.946
CycE	0.586	E2F	0.954	CycD-CDK2-p21	0.915
CycA	0.525	CycD-CDK2-p21	0.904	CycE-CDK2-p21	0.863
CycD-CDK4/6	0.474	CycE-CDK2-p21	0.888	CycD-CDK2-p16	0.835
CycD-CDK2-p21	0.464	CycA-CDK2	0.86	CycD-CDK2-p27	0.763
CycA-CDK2	0.42	CycA	0.86	CycA-CDK2	0.744
E2F	0.419	CycE-CDK2	0.275	CycE	0.566
p21	0.353	CycE-CDK2-p27	0.267	CycA	0.505
CycE-CDK2-p27	0.324	CDK2	0.256	CycD-CDK4/6	0.482
CycE-CDK2-p21	0.287	CycD-CDK2-p27	0.242	E2F	0.473
CycA-CDK2-p27	0.23	E2F-Rbpp	0.178	CycA-CDK2-p27	0.272
CycE-CDK2	0.22	CycA-CDK2-p27	0.174	CDK2	0.22
CycD	0.211	p27	0.141	CycD	0.22
Rbpppp	0.185	CycD	0.118	Rbpppp	0.188
CDK4/6	0.111	CycD-CDK4/6	0.101	CDK4/6	0.115
CycA-CDK2-p21	0.102	CycD-CDK2-p16	0.057	p27	0.087
CDK2	0.098	E2F-Rb	0.053	E2F-Rb	0.083
E2F-Rb	0.083	Rbpppp	0.042	CycE-CDK2	0.068
p27	0.037	Skp2	0.015	CycE-CDK2-p27	0.061
Skp2	0.011	CDK4/6	0.013	Skp2	0.015
p16	0.01	p16	0.005	p16	0.011
Rb	0.005	Rb	0.004	Rb	0.007

Table S2: Crosstalk in the cell cycle coupled to p53, to NF-κB and both p53 and NF-κB with double coupling strength. Each species of the cell cycle is assigned a *D* value (Equation (4)) in the interval [0,1] by Procedure B (Methods), where larger values indicate greater crosstalk. Values less than 0.1 correspond to amounts of crosstalk which are difficult to discern in the time and frequency plots.

NF-κB – cell cycle	*D*	p53 – cell cycle	*D*	p53 – NF-κB – cell cycle	*D*
E2F-Rbpp	0.943	p21	0.998	p21	0.998
CycD-CDK2-p16	0.932	CycD-CDK2-p21	0.996	CycD-CDK2-p21	0.998
CycD-CDK2-p27	0.856	CycE	0.995	E2F	0.993
CycD-CDK4/6	0.781	E2F	0.994	CycE	0.993
CycA	0.631	CycA-CDK2	0.994	CycA-CDK2	0.99
CycD-CDK2-p21	0.613	CycE-CDK2-p21	0.99	CycA-CDK2-p21	0.98
CycA-CDK2	0.572	CycA-CDK2-p21	0.974	CycE-CDK2-p21	0.976
CycE-CDK2-p27	0.53	CycA	0.958	CycA	0.952
CycE	0.513	CycA-CDK2-p27	0.753	E2F-Rbpp	0.945
p21	0.513	p27	0.469	CycD-CDK2-p16	0.929
CycD	0.451	CycE-CDK2	0.455	CycD-CDK2-p27	0.872
CycE-CDK2-p21	0.431	CycE-CDK2-p27	0.392	CycA-CDK2-p27	0.828
CycA-CDK2-p27	0.295	CycD-CDK2-p27	0.381	CycD-CDK4/6	0.816
E2F	0.232	E2F-Rbpp	0.369	CycD	0.52
CDK4/6	0.227	CycD	0.33	CycE-CDK2	0.399
CycE-CDK2	0.227	CycD-CDK4/6	0.306	p27	0.36
Rbpppp	0.188	CDK2	0.263	CDK2	0.262
CycA-CDK2-p21	0.149	CycD-CDK2-p16	0.138	CDK4/6	0.231
CDK2	0.086	E2F-Rb	0.08	Rbpppp	0.19
E2F-Rb	0.083	Rbpppp	0.042	CycE-CDK2-p27	0.162
p27	0.058	CDK4/6	0.016	E2F-Rb	0.083
p16	0.009	Skp2	0.015	p16	0.01
Rb	0.008	Rb	0.009	Rb	0.008
Skp2	0.005	p16	0.005	Skp2	0.007

Table S3: Crosstalk in the cell cycle coupled to p53, to NF-κB and both p53 and NF-κB with ten times coupling strength. Each species of the cell cycle is assigned a *D* value (Equation(4)) in the interval [0,1] by Procedure B (Methods), where larger values indicate greater crosstalk. Values less than 0.1 correspond to amounts of crosstalk which are difficult to discern in the time and frequency plots.

The effect of stochasticity on independent networks

Table S4 quantifies the differences between the fully stochastic and quasi-deterministic models in isolation. Although there are some species with apparently significant differences, the coupling species (p53a and NF-κBn) show minimal stochasticity and the effect on crosstalk was found to be negligible. Some interesting behavioural differences are noted in the main text.

p53	*D*	NF-κB	*D*	Cell cycle	*D*	Cell cycle	*D*
p53i	0.025	nIkB-NF-kB	0.46	CycE-CDK2-p27	0.94	CycA-CDK2-p21	0.017
p53a	0.01	IkBn	0.34	CycE-CDK2-p21	0.863	CycD	0.015
Mdm2	0.01	IkB	0.11	CycD-CDK2-p21	0.685	CycA-CDK2	0.012
I	0.01	IKK-IkB	0.055	CycE-CDK2	0.455	Skp2	0.012
S	0.01	NF-kB	0.035	p21	0.122	Rbpppp	0.011
		KIkB-NF-kB	0.03	CycD-CDK4/6	0.073	Rb	0.008
		IkB-NF-kB	0.025	CycE	0.072	CycD-CDK2-p27	0.007
		IkBt	0.015	E2F	0.053	CDK2	0.006
		IKK	0.015	E2F-Rbpp	0.034	p16	0.006
		NF-kBn	0.01	CycA	0.02	CDK4/6	0.003
				CycA-CDK2-p27	0.019	p27	0.003
				CycD-CDK2-p16	0.019	E2F-Rb	0.003

Table S4: Differences between reaction-based and quasi-deterministic models in the absence of coupling. Each species is assigned a *D* value (Equation (4)) in the interval [0,1] by Procedure B (Methods), where larger values indicate greater difference. Values less than 0.1 correspond to minimal visible stochasticity in the time courses. Coupling species p53a and NF-κBn have very low stochasticity, so the cell cycle is minimally affected by the stochasticity in the perturbing systems.

Supplementary methods

The following subsections describe the construction of the system of networks and simulation models used in the main text. The three networks are given as independent models, with the coupling reactions itemised separately. The rate constants and initial numbers of molecules are common to both the stochastic and quasi-deterministic models. The following general assumptions were made: earlier studies revealed oscillatory NF-κB activity in cells lacking IκB β & ε [1] and biphasic dynamics in cells in which NF-κB-inducible IκBα was over-expressed [2]; induced expression of IκBε also gives rise to an oscillatory NF-κB signal that is out of phase with IκBα-induced oscillations [3] and which helps to keep the late phase of TNFα- and IL1-α-induced NF-κB activity steady [4]; possibility that oscillations are a trade-off against rapid response to inflammatory signals and the necessity of additional feedback to provide steady supply of active NF-κB, which might arguably be present in other linking pathways – e.g. p53-MDM2; in contrast to some other models that focus mainly on G1/S-phase transition control [5-9], this model is only interested in key processes involving data sets previously published (which strengthens the idea that cell cycle process can also be induced by NF-κB active proteins).

NF-κB system

We and others have previously re-constructed a computational model to explain NF-κB activation events following IKK activation by TNFα stimulation [2, 10]. That model, which represented the most studied aspect of the NF-κB pathway, comprises NF-κB, canonical IκB α/β/ε and IKK. Both the IKK and NF-κB are represented as singular species (without separate descriptions for the IKKα/β heterodimer and its scaffold protein IKKγ). Their synthesis, degradation, cellular localisation and interactions were calculated using a deterministic method. The key processes modelled included: mRNA transcription and protein translation of NF-κB Inhibitor protein IκB (IκBα, IκBβ, IκBε); the inter compartment transport of IκB (IκBα, IκBβ, IκBε), NF-κB and their complexes; formation of protein complexes; catalytic activation of canonical and alternative IKK; catalytic degradation of the NF-κB Inhibitor proteins due to IKK-induced phosphorylation and subsequent ubiquitination.

Cell cycle system

Many groups have reported the construction of the mammalian cell cycle models, the most recent being [11, 12]. Works on models of yeast cell cycle are more advanced and incorporate explicit representation of cell mass and cell growth. For the mammalian cell cycle model used here, we have

not included cell growth, as we were only interested in the events leading to the G1/S transition phase, the point where NF-κB and p53 signal transduction events are active the most. The model comprises: G1 phase; inhibition of cell cycle activity as a result of Rb binding to E2F. For the activation of the cell cycle and thus the transition phase from G1 to S, Rb phosphorylation by the CDKs (CDK4/6, CDK2) is necessary, which results in the activation and release of E2F. However, the CDKs have their inhibitory counterparts (p16, p21 and p27). The model receives as input a signal from the NF-κB signal transduction pathway for the synthesis of CycD1, which quickly forms a complex with CDK4/6 to become the active form CycD-CDK4/6. This complex can further bind to their inhibitory counterpart forming the following; CycD-CDK4/6-p16, CycD-CDK4/6-p21 and CycD-CDK4/6-p27, out of which formation of CycD-CDK4/6-p16 inhibits its activity. Hypo-phosphorylation of the bound Rb (Rb-E2F) by complex formation of CycD-CDK4/6 releases E2F. Free E2F is involved in CycE, CycA and Skp2 gene transcription. Free CycE and CycA form complexes with CDK2 to form active compounds. CycE-CDK2 and CycA-CDK2 can also act as a further phosphorylating factor to bound Rb (Rb-E2F), releasing more E2F. CycE and CycA complexes also bind to their inhibitory counterparts forming the following: CycE-CDK2-p27, CycE-CDK2-p21, CycA-CDK2-p27, CycA-CDK2-p21; and is mainly described by mass balance equations. The parameter values have been chosen to quantitatively and qualitatively represent the phases of the cell cycle of interest. The degradation of the components were also accounted for in the model.

DNA damage transduction system

Stimuli such as DNA damage can activate both the NF-κB and the p53 pathways [13]. While p53 induces cell-cycle arrest or cell death in response to these treatments, the contribution of NF-κB to cell fate is more complex, and pathways in which it either antagonizes or cooperates with p53 have been described. NF-κB-mediated negative regulation of p53 can contribute to tumorigenesis and has been shown to operate at a number of levels. The delay oscillator describe by Geva-Zatorsky *et. al* [14] was the model of choice for the p53 system.

Stochastic delay differential equations

Unlike the cell cycle and NF-κB models, which are described by standard chemical and enzymatic reactions that can be straight-forwardly converted into the stochastic domain, the original p53 model is constructed around delay differential equations (DDEs) and requires special consideration when simulating stochastically. DDEs allow the current rate of a reaction to be dependent on the state of the system at some time in the past, abstracting potentially very complex (unknown) mechanisms into a combination of delays. Stochastic simulations based on a variant of the Gillespie algorithm [15] treat the evolution of the system as a Markov process, such that the current rate of a reaction is only dependent on the *current* state of the system. To incorporate a model based on DDEs into a stochastic simulation of this kind, it is also necessary to store the past states of the system. We thus implemented a function in our simulation software which returns the number of molecules of species X at τ time units in the past: delay(X,τ). Given that the time course of a stochastic simulation consists of the numbers of molecules of different species recorded at irregular time points 0, t_1, t_2, ... etc., where $0 < t_1 < t_2 < ...$, the amount of species X at time t can be described by the sequence X_0, Xt_1, Xt_2, ..., etc. At time t, the value returned by delay(X,τ) is then Xt_i, where i is the maximum value that satisfies $t_i \leq t-\tau$. If $t < \tau$ (as may happen at the beginning of a simulation), delay(X,τ) returns X_0. This algorithm is consistent with the standard deterministic interpretation of delay differential equations and guarantees that for *any* specified initial state and past state corresponding to the delay used, the magnitude and direction of the average rate of leaving the state in the stochastic and quasi-deterministic models is *identical* (allowing for the conversion from concentration to numbers of molecules) to that for the deterministic case.

Model naming convention

For the purposes of simulation we used simplified names of the chemical species. The following table maps the names used in the text to the names used in the models.

Text	Model
p53i	p53i
p53a	p53a
Mdm2	Mdm2
I	I
S	S
IkBn-NF-kBn	nIkBNFkB
IkBn	IkBn
IkB	IkB
IKK-IkB	IKKIkB
NF-kB	NFkB
IKK-IkB-NF-kB	KIkBNFkB
IkB-NF-kB	IkBNFkB
IkBt	IkBt
IKK	IKK
NF-kBn	NFkBn

Text	Model
CycA-CDK2-p21	CycACDp21
CycA-CDK2-p27	CycACDp27
CycA-CDK2	CycACDK2
CycD-CDK4/6	CycDCDK46
CycD-CDK2-p27	CycDCDp27
CycD-CDK2-p16	CycDCDp16
CycD-CDK2-p21	CycDCDp21
CycE-CDK2	CycECDK2
CycE-CDK2-p27	CycECDp27
CycE-CDK2-p21	CycECDp21
E2F-Rbpp	E2FRbpp
E2F-Rb	E2FRb

Text	Model
CycA	CycA
CycD	CycD
CycE	CycE
CDK4/6	CDK46
Skp2	Skp2
Rbpppp	Rbpppp
Rb	Rb
CDK2	CDK2
p16	p16
p27	p27
p21	p21
E2F	E2F

Stochastic (reaction-based) models

The following models are based on standard chemical reactions of the kind A + B → C + D, where a single reaction event simultaneously consumes molecules of A and B while producing molecules of C and D. Where not otherwise stated, the rates of reactions are calculated using the given constants and the assumption of mass action. An explicit rate function to generate the reaction *propensity* [15] is given for reactions where this does not apply. The function delay(·) is defined above. The symbol Ø is used to denote an arbitrary source or sink in creation and degradation reactions, respectively.

p53

Reaction	Rate constant / function
p53i + Mdm2 → Mdm2	kap53i
Ø → p53i	kbp53i
p53a + Mdm2 → Mdm2	kap53a
p53i → p53a	$w\times(S^n/(S^n+Ts))\times p53i$
ARF + p53a → 2 p53a	R46
Mdm2 → Ø	kaMdm2
Mdm2 + ARF → Ø	R48
Ø → Mdm2	kbMdm2×delay(p53a,tau)
I → Ø	kai
Ø → I	kbi×(delay(p53a,tau)+delay(p53i,tau))
ARF → Ø	R47
Ø → ARF	R45a
S + I → I	kas
Ø → S	kbs×e

Cell cycle

Reaction	Rate constant
CycDCDK46 → CDK46	R1
CycDCDK46 + p16 → CycDCDp16	R29
CycDCDK46 + p27 → CycDCDp27	R6
CycDCDK46 → CDK46 + CycD	R21b
CycD + CDK46 → CycDCDK46	R21a
CDK46 → Ø	R32
CycACDK2 + E2F → CycACDK2	R15
Ø → E2F	R43
E2F → E2F + E2F	R42
CycE → Ø	R26
E2F → CycE + E2F	R2
CycECDK2 → CDK2	R3

CycECDK2 → CDK2 + CycE	R24b
CDK2 + CycE → CycECDK2	R24a
CDK2 + CycA → CycACDK2	R25a
CDK2 → Ø	R33
CycA → Ø	R27
E2F → CycA + E2F	R4
CycACDK2 → CDK2	R5
CycACDK2 → CycA + CDK2	R25b
p27 + CycECDK2 → CycECDp27	R7
p27 + CycACDK2 → CycACDp27	R8
Ø → p27	R20
CycECDp27 + Skp2 → Skp2 + CycECDK2	R9
CycACDp27 + Skp2 → Skp2 + CycACDK2	R10
Skp2 → Ø	R34
Ø → Skp2	R31
Rb → Ø	R18
Rb + E2F → E2FRb	R11
Ø → Rb	R17
Rbpppp → Rb	R16
CycDCDK46 + E2FRb → E2FRbpp + CycDCDK46	R12
CycDCDp27 + E2FRb → E2FRbpp + CycDCDp27	R13
CycDCDp21 + E2FRb → E2FRbpp + CycDCDp21	R41
E2FRbpp + CycECDK2 → CycECDK2 + Rbpppp + E2F	R14
CycDCDp16 → p16	R19
p16 → Ø	R23
Ø → p16	R28
CycD → Ø	R22
E2F → CycD + E2F	R44
Ø → CycD	R30a
p21 + CycDCDK46 → CycDCDp21	R35a
p21 + CycECDK2 → CycECDp21	R36a
p21 + CycACDK2 → CycACDp21	R37a
Ø → p21	R40a
CycDCDp21 → p21 + CycDCDK46	R35b
CycECDp21 → p21 + CycECDK2	R36b
Skp2 + CycECDp21 → CycECDK2 + Skp2	R38
CycACDp21 → p21 + CycACDK2	R37b
Skp2 + CyCACDp21 → CycACDK2 + Skp2	R39

NF-κB

Reaction	Rate constant / function
IkB → Ø	kdeg1
IkB → IkBn	ktp1
IkB + NFkB → IkBNFkB	la4
IkBt → IkB + IkBt	ktr1
IkBn → IkB	ktp2
IkBn + NFkBn → nIkBNFkB	la4
IkBn → Ø	kdeg1
nIkBNFkB → IkBn + NFkBn	kd4
nIkBNFkB → IkBNFkB	k2
nIkBNFkB → NFkBn	kdeg5
IkBNFkB → nIkBNFkB	k3
IkBt → Ø	ktr3
Ø → IkBt	tr2a
Ø → IkBt	$tr2\times(NFkBn)^{h}$
IkBNFkB → IkB + NFkB	kd4
IkBNFkB → NFkB	kdeg4
IKK + IkB → IKKIkB	la1
IKK + IkBNFkB → KIkBNFkB	la7
IKK → Ø	k02
IKKIkB → IKK + IkB	kd1
IKKIkB → IKK	kr1
IKKIkB + NFkB → KIkBNFkB	la4
KIkBNFkB → IKK + IkBNFkB	kd2
KIkBNFkB → IKKIkB + NFkB	kd4
KIkBNFkB → NFkB + IKK	kr4
NFkB → NFkBn	k1
NFkBn → NFkB	k01

Coupling reactions

Reaction	Rate constant / function
Ø → CycD	$R30b\times(NFkBn)^{h}$
p53a → p21 + p53a	R40b

Quasi-deterministic (combined production / consumption) models

The following models are based on quasi-deterministic ‘reactions’, where a single reaction event is an unsynchronised production or consumption of a single molecule of a given species. The *propensity* [15] of a quasi-deterministic reaction is given by an explicit rate function that is the signed sum of the propensities of creation and consumption reactions.

p53

Species	Combined rate function of production and consumption
p53i	kbp53i-kap53i×Mdm2×p53i-w×(S^n/(S^n+Ts))×p53i
p53a	w×(S^n/(S^n+Ts))×p53i-kap53a×Mdm2×p53a+R46×p53a×ARF
Mdm2	kbMdm2×delay(p53a,tau)-kaMdm2×Mdm2-R48×ARF×Mdm2
I	kbi×(delay(p53a,tau)+delay(p53i,tau))-kai×I
ARF	R45a-R46×p53a×ARF-R47×ARF-R48×ARF×Mdm2
S	kbs×e-kas×I×S

NF-κB

Species	Combined rate function of production and consumption
IkB	-(kdeg1+ktp1)×IkB+ktp2×IkBn+ktr1×IkBt+kd4×IkBNFkB+kd1×IKKIkB-la1×IkB×IKK -la4×IkB×NFkB
IkBn	ktp1×IkB+kd4×nIkBNFkB-ktp2×IkBn-la4×IkBn×NFkBn-kdeg1×IkBn
nIkBNFkB	-kd4×nIkBNFkB-k2×nIkBNFkB-kdeg5×nIkBNFkB+k3×nIkBNFkB+la4×IkBn×NFkBn
IkBt	tr2a-ktr3×IkBt+tr2×$(NFkBn)^h$
IkBNFkB	k2×nIkBNFkB-kd4×IkBNFkB-kdeg4×IkBNFkB-la7×IkBNFkB×IKK-k3×nIkBNFkB +kd2×KIkBNFkB+la4×IkB×NFkB
IKK	-la1×IkB×IKK-la7×IkBNFkB×IKK-k02×IKK+kd1×IKKIkB+kr1×IKKIkB+kd2×KIkBNFkB +kr4×KIkBNFkB
IKKIkB	la1×IkB×IKK-kd1×IKKIkB-kr1×IKKIkB+kd4×KIkBNFkB-la4×IKKIkB×NFkB
KIkBNFkB	la7×IkBNFkB×IKK-kd2×KIkBNFkB-kd4×KIkBNFkB-kr4×KIkBNFkB+la4×IKKIkB×NFkB
NFkB	kd4×IkBNFkB+kdeg4×IkBNFkB+kd4×KIkBNFkB+kr4×KIkBNFkB+k01×NFkBn-k1×NFkB -la4×IkB×NFkB-la4×IKKIkB×NFkB
NFkBn	kd4×nIkBNFkB+k1×NFkB+k3×nIkBNFkB-k01×NFkBn-la4×IkBn×NFkBn

Cell cycle

Species	Combined rate function of production and consumption
CycDCDK46	-R1×CycDCDK46-R29×CycDCDK46×p16-R6×CycDCDK46×p27-R35a×CycDCDK46×p21 -R21b×CycDCDK46+R35b×CycDCDp21+R21a×CycD×CDK46
CDK46	R1×CycDCDK46+R21b×CycDCDK46-R21a×CycD×CDK46-R32×CDK46
E2F	R43+R42×E2F+R14×E2FRbpp×CycECDK2-R11×Rb×E2F-R15×E2F×CycACDK2
CycE	R2×E2F+R24b×CycECDK2-R24a×CycE×CDK2-R26×CycE
CycECDK2	-R3×CycECDK2-R7×CycECDK2×p27-R36a×CycECDK2×p21-R24b×CycECDK2 +R36b×CycECDp21+R24a×CycE×CDK2+R9×CycECDp27×Skp2+R38×CycECDp21×Skp2
CDK2	R3×CycECDK2+R5×CycACDK2+R24b×CycECDK2+R25b×CycACDK2 -R24a×CycE×CDK2-R25a×CycA×CDK2-R33×CDK2
CycA	R4×E2F+R25b×CycACDK2-R25a×CycA×CDK2-R27×CycA
CycACDK2	-R5×CycACDK2-R8×CycACDK2×p27-R37a×CycACDK2×p21-R25b×CycACDK2 +R37b×CycACDp21+R25a×CycA×CDK2+R10×CycACDp27×Skp2 +R39×CycACDp21×Skp2
p27	R20-R6×CycDCDK46×p27-R7×CycECDK2×p27-R8×CycACDK2×p27
CycDCDp27	R6×CycDCDK46×p27
CycECDp27	R7×CycECDK2×p27-R9×CycECDp27×Skp2
CycACDp27	R8×CycACDK2×p27-R10×CycACDp27×Skp2
Skp2	R31-R34×Skp2
Rb	R17+R16×Rbpppp-R18×Rb-R11×Rb×E2F
E2FRb	R11×Rb×E2F-R12×E2FRb×CycDCDK46-R13×E2FRb×CycDCDp27 -R41×E2FRb×CycDCDp21
E2FRbpp	R12×E2FRb×CycDCDK46+R13×E2FRb×CycDCDp27 +R41×E2FRb×CycDCDp21-R14×E2FRbpp×CycECDK2
Rbpppp	R14×E2FRbpp×CycECDK2-R16×Rbpppp
CycDCDp16	R29×CycDCDK46×p16-R19×CycDCDp16
p16	R28+R19×CycDCDp16-R29×CycDCDK46×p16-R23×p16
CycD	R44×E2F+R21b×CycDCDK46-R21a×CycD×CDK46-R22×CycD+R30a
p21	R40a-R35a×CycDCDK46×p21-R36a×CycECDK2×p21-R37a×CycACDK2×p21 +R35b×CycDCDp21+R36b×CycECDp21+R37b×CycACDp21
CycDCDp21	R35a×CycDCDK46×p21-R35b×CycDCDp21
CycECDp21	R36a×CycECDK2×p21-R36b×CycECDp21-R38×CycECDp21×Skp2
CycACDp21	R37a×CycACDK2×p21-R37b×CycACDp21-R39×CycACDp21×Skp2

Coupling reactions

Species	Combined rate function of production and consumption with coupling
CycD	R44×E2F+R21b×CycDCDK46-R21a×CycD×CDK46-R22×CycD+R30a+R30b×$(NFkBn)^{h}$
p21	R40a-R35a×CycDCDK46×p21-R36a×CycECDK2×p21-R37a×CycACDK2×p21 +R35b×CycDCDp21+R36b×CycECDp21+R37b×CycACDp21+R40b×p53a×p21

Rate and other constants

The following constants are common to both sets of models. The reaction rates are derived from models based on concentration, hence *alpha* has units of $l\ mol^{-1}$ and is the constant which is used to

convert these into numbers of molecules. A nominal value of *alpha* = 100000 was chosen, based on an estimate of nuclear volume. Rate constants defined as a value divided by *alpha* generally correspond to bimolecular reactions of the kind A + B → ... and have units $mol^{-1} l\ minutes^{-1}$, while rate constants defined as a value multiplied by *alpha* generally correspond to creation reactions of the kind Ø → ... and have units $mol\ l^{-1} minutes^{-1}$. Reaction rate constants which are not a function of *alpha* generally correspond to simple degradation reactions of the kind A → ... and have units $minutes^{-1}$. There were no homodimerisation reactions.

Name	Value	Name	Value	Name	Value
alpha	100000	R8	7.0×10^{-2}/alpha	R35b	5.0×10^{-3}
h	2	R9	0.225/alpha	R36a	1.0×10^{-2}/alpha
kdeg1	0.16	R10	2.5×10^{-3}/alpha	R36b	1.75×10^{-4}
ktp1	0.018	R11	5.0×10^{-5}/alpha	R37a	7.0×10^{-2}/alpha
ktp2	0.012	R12	1.0×10^{-4}/alpha	R37b	1.75×10^{-4}
ktr1	0.2448	R13	1.0×10^{-2}/alpha	R38	0.225/alpha
kd4	0.00006	R14	0.073/alpha	R39	2.5×10^{-3}/alpha
la1	0.1776/alpha	R15	0.022/alpha	R40a	5.0×10^{-5}×alpha
kd1	0.000888	R16	5.0×10^{-8}	R40b	1.0×10^{-3}
la4	30/alpha	R17	5.0×10^{-5}×alpha	R41	1.0×10^{-2}/alpha
k2	0.552	R19	5.0×10^{-2}/alpha	R42	1.0×10^{-4}
k3	0.00006	R20	1.0×10^{-4}×alpha	R43	5.0×10^{-5}×alpha
tr2a	0.000090133×alpha	R21a	2.0×10^{-3}/alpha	R44	3.0×10^{-4}
ktr3	0.020733	R21b	8.0×10^{-3}	R45a	8.0×10^{-5}×alpha
tr2	$0.5253/alpha^{(h-1)}$	R22	7.5×10^{-3}	R45b	0.008
kdeg4	0.00006	R23	5.0×10^{-3}	R46	2.333×10^{-5}/alpha
kdeg5	0.00006	R24a	8.0×10^{-3}/alpha	R47	0.01167
la7	6.06/alpha	R24b	3.9×10^{-3}	R48	1.167×10^{-5}/alpha
kd2	0.095	R25a	8.0×10^{-3}/alpha	kbp53i	0.015×alpha
kr1	0.012	R25b	4.0×10^{-3}	kbMdm2	0.01667
kr4	0.22	R26	2.5×10^{-3}	kap53i	2.333/alpha
k1	5.4	R27	5.0×10^{-4}	kaMdm2	0.01167
k01	0.0048	R28	2.0×10^{-4}×alpha	tau	80
k02	0.0072	R29	5.0×10^{-4}/alpha	kap53a	0.02333/alpha
R1	5.0×10^{-6}	R30a	0.004×alpha	kas	0.045/alpha
R2	4.5×10^{-3}	R30b	$0.9961/alpha^{(h-1)}$	kbi	0.01667
R3	5.0×10^{-3}	R31	5.0×10^{-4}×alpha	kai	0.01167
R4	2.5×10^{-3}	R32	8.0×10^{-4}	kbs	0.015×alpha
R5	5.0×10^{-4}	R33	8.0×10^{-4}	e	1
R6	5.0×10^{-4}/alpha	R34	9.0×10^{-4}	n	4
R7	1.0×10^{-2}/alpha	R35a	5.0×10^{-4}/alpha	w	11.665
				Ts	$1\times alpha^{n}$

Initial numbers of molecules

The following table contains the initial numbers of molecules used by all the simulation models. Note that the values are either 0 or some number (an initial concentration in units of *mol* l^{-1}) multiplied by *alpha*, the global constant used to specify the number of molecules in the system.

Species	Amount
p53i	0
p53a	0.1×alpha
Mdm2	0.15×alpha
I	0.1×alpha
S	0
ARF	0
IkB	0
IkBn	0
nIkBNFkB	0
IkBt	0
IkBNFkB	0.2×alpha
IKK	0.2×alpha
IKKIkB	0
KIkBNFkB	0
NFkB	0
NFkBn	0.025×alpha
CycDCDK46	0
CDK46	5.0×alpha
E2F	0
CycE	0

Species	Amount
CycECDK2	0
CDK2	2.0×alpha
CycA	0
CycACDK2	0
p27	1.0×alpha
CycDCDp27	0.001×alpha
CycECDp27	0
CycACDp27	0
Skp2	1.0×alpha
Rb	1.0×alpha
E2FRb	1.95×alpha
E2FRbpp	1.0×10^{-3}×alpha
Rbpppp	1.02×alpha
CycDCDp16	1.0×10^{-5}×alpha
p16	1.0×alpha
CycD	0
p21	0
CycDCDp21	0
CycECDp21	0
CycACDp21	0

Supplementary example: a stochastic model of the eukaryotic cell cycle

The presented technique of frequency domain analysis can be particularly useful and revealing when applied to stochastic simulations of chemical systems containing one or more species in low copy numbers. The apparent behaviour in such simulation time courses may be very noisy, yet the underlying average behaviour will nevertheless be obvious to human observers. Frequency domain analysis is a means to formalise the perceived behaviour. Figure S7 illustrates the differences between stochastic and deterministic simulations, using as example the generic model of the eukaryotic cell cycle in [18]. Table S5 describes the stochastic model, extracted from the ODEs, comprising elemental reactions using mass action kinetics, enzymatic reactions with arbitrary kinetics and mass balance equations. Parameters are chosen to represent budding yeast and the species names are those used in [18]. The deterministic model exhibits limit cycle oscillation,

making it conceivable to run arbitrarily long stochastic simulations and so arbitrarily define the resolution of the frequency domain analysis. This is not in general guaranteed: when a continuous deterministic model is discretised and made stochastic (or quasi-deterministic, as described above), it may contain states which have a non-zero probability of being reached but from which the system cannot exit. These *absorbing states* may exist in reality or may be unforeseen artefacts of the ODE approximation of reality, hence the validity of stochastic models created from deterministic systems containing arbitrary simplifications and abstractions is sometimes questioned. Such questions may be answered by the presented methodology.

Figure S7A shows a typical time course of CycBT (black) in the stochastic model of the budding yeast cell cycle, exhibiting variable amplitude and phase. In red is the result of averaging 800 such time series: random phase shifts between independent simulation runs cause average oscillatory behaviour to decay with time and for the oscillatory waveform to become more sinusoidal; the average trajectory gets closer to the long term mean number of molecules of CycBT (grey line). Figure S7B compares frequency spectra of deterministic (black), quasi-deterministic (blue) and fully stocahastic (red) models of the budding yeast cell cycle. The deterministic spectrum (created from a single time course of 10000 minutes sampled at 5 minute intervals) is clearly ‘spiky’ in nature, with many evident high frequency components and apparent numerical artefacts. By contrast, the average stochastic frequency spectrum (red) contains only four discernable low frequency peaks that are relatively rounded. The spectrum of the quasi-deterministic simulation appears closer to the fully stochastic than to the deterministic, however it contains three more discernable peaks and at higher frequencies it follows more closely the trend of the deterministic spectrum. The peaks of the stochastic and quasi-deterministic spectra apparently align with peaks in the deterministic spectrum, suggesting that the three systems have the same average primary mode of oscillation, however this alignment between models is not in general guaranteed. In each case the spectral value at zero frequency corresponds to the long term mean of the time series.

A

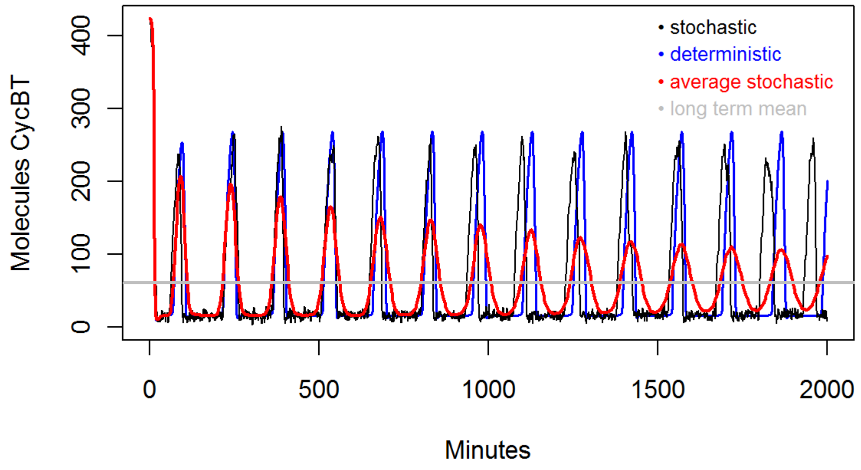

B

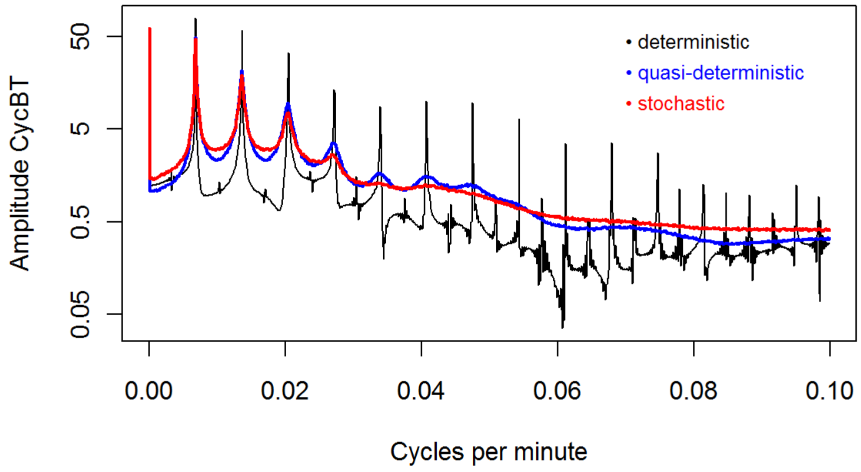

Figure S7: Time and frequency domain behaviour of CycBT in generic eukaryotic cell cycle
A Typical (black) and average (red) time series of CycBT in the stochastic version of the generic model of the eukaryotic cell cycle of Table S5. As a result of random phase shifts between simulation runs, oscillatory behaviour in the average trace decays with time and hovers around the long term average number of molecules of CycBT (grey line). **B** Average frequency distribution of CycBT in stochastic (red), quasi-deterministic (blue) and determinist (black) models. The deterministic spectrum is clearly more 'spiky' than the stochastic spectra and has many high frequency components that are apparently 'lost in the noise' of the other models. The average time course was created from 800 simulation traces of 4000 minutes sampled at 2 minute intervals. The average frequency spectra were created from 800 traces of 10000 minutes sampled at 5 minute intervals. The deterministic spectrum was created from a single trace of 10000 minutes sampled at 5 minute intervals.

Elemental reaction	Mass action rate constant		Other constants	
Ø → CycBT	k1	0.04×alpha	k3.1	1×alpha
CycBT → Ø	k2.1	0.04	k3.2	10
Cdh1 + CycBT → Cdh1	k2.2	1/alpha	k4	35
CycBT + Cdc20A → Cdc20A	k2.3	1/alpha	k4.1	2
Ø → Cdc20T	k5.1	0.005×alpha	k5.2	0.2×alpha
Cdc20T → Ø	k6	0.1	k7	1
Cdc20A → Ø	k6	0.1	k8	0.5
IE → Ø	k10	0.02	k9	0.1/alpha
Ø → CKIT	k11	1×alpha	k15.1	1.5×alpha/beta
CKIT → Ø	k12.1	0.2	k15.2	0.05
CKIT + SK → SK	k12.2	50/alpha	k16.1	1×alpha
CKIT + CycB → CycB	k12.3	100/alpha	k16.2	3
Ø → SK	k13.1	0×alpha	J3, J4	0.04×alpha
TF → SK + TF	k13.2	1	J5	0.3×alpha
SK → Ø	k14	1	J7, J8	0.001×alpha
			J15, J16	0.01×alpha
			mu	0.005
			Mstar	10×beta
			Kdiss	0.001×alpha

Enzymatic reaction	Reaction kinetics
Ø → Cdh1	(k3.1+k3.2×Cdc20A)×(alpha-Cdh1)/(J3+alpha-Cdh1)
Cdh1 → Ø	(k4.1×SK+k4×CycB)×Cdh1/(J4+Cdh1)
Ø → Cdc20T	k5.2×CycB^4/(J5^4+CycB^4)
Ø → Cdc20A	k7×IE×(Cdc20T-Cdc20A)/(J7+Cdc20T-Cdc20A)
Cdc20A → Ø	k8×Mad1×Cdc20A/(J8+Cdc20A)
Ø → IE	k9×(alpha-IE)×CycB
Ø → TF	(k15.1×M+k15.2×SK)×(alpha-TF)/(J15+alpha-TF)
TF → Ø	(k16.1+k16.2×CycB)×TF/(J16+TF)
Ø → M	mu×M×(1-M/Mstar)

Mass balance equations
BB = CycBT+CKIT+Kdiss
CycB = (1-2×CKIT/(BB+sqrt(BB^2-4×CycBT×CKIT)))×CycBT×M/beta

Table S5: Stochastic model of the generic eukaryotic cell cycle. The ODE model of [18] was resolved into elemental reactions with mass action kinetics and enzymatic reactions having arbitrary kinetic laws. A constant of *alpha* = 424 $l\ mol^{-1}$ was used to convert initial concentrations and rate constants to numbers of molecules. To discretise the cell growth, a mass granularity constant of *beta* = 1000 was adopted.

References

1. **The IκB-NF-κB signaling module: temporal control and selective gene activation/ Supporting Material.** [http://www.sciencemag.org/cgi/data/298/5596/1241/DC1/2]
2. Nelson DE, Ihekwaba AE, Elliott M, Johnson JR, Gibney CA, Foreman BE, Nelson G, See V, Horton CA, Spiller DG *et al*: **Oscillations in NF-κB signaling control the dynamics of gene expression**. *Science* 2004, **306**(5696):704-708.
3. Kearns JD, Basak S, Werner SL, Huang CS, Hoffmann A: **IκBepsilon provides negative feedback to control NF-κB oscillations, signaling dynamics, and inflammatory gene expression**. *J Cell Biol* 2006, **173**(5):659-664.
4. Ihekwaba AE, Wilkinson SJ, Waithe D, Broomhead DS, Li P, Grimley RL, Benson N: **Bridging the gap between in silico and cell-based analysis of the nuclear factor-κB signaling pathway by in vitro studies of IKK2**. *Febs J* 2007, **274**(7):1678-1690.
5. Obeyesekere MN, Herbert JR, Zimmerman SO: **A model of the G1 phase of the cell cycle incorporating cyclin E/cdk2 complex and retinoblastoma protein**. *Oncogene* 1995, **11**(6):1199-1205.
6. Obeyesekere MN, Tucker SL, Zimmerman SO: **A model for regulation of the cell cycle incorporating cyclin A, cyclin B and their complexes**. *Cell Prolif* 1994, **27**(2):105-113.
7. Kohn KW: **Functional capabilities of molecular network components controlling the mammalian G1/S cell cycle phase transition**. *Oncogene* 1998, **16**(8):1065-1075.
8. Aguda BD, Tang Y: **The kinetic origins of the restriction point in the mammalian cell cycle**. *Cell Prolif* 1999, **32**(5):321-335.
9. Qu Z, Weiss JN, MacLellan WR: **Regulation of the mammalian cell cycle: a model of the G1-to-S transition**. *Am J Physiol Cell Physiol* 2003, **284**(2):C349-364.
10. Ihekwaba AEC, Broomhead DS, Grimley RL, Benson N, Kell DB: **Sensitivity analysis of parameters controlling oscillatory signalling in the NF-κB pathway: the roles of IKK and IκBα**. *Systems Biology* 2004, **1**(1):93-103.
11. Iwamoto K, Tashima Y, Hamada H, Eguchi Y, Okamoto M: **Mathematical modeling and sensitivity analysis of G1/S phase in the cell cycle including the DNA-damage signal transduction pathway**. *Biosystems* 2008, **94**(1-2):109-117.
12. Ling H, Kulasiri D, Samarasinghe S: **Robustness of G1/S checkpoint pathways in cell cycle regulation based on probability of DNA-damaged cells passing as healthy cells**. *Biosystems* 2010, **101**(3):213-221.
13. Perkins ND: **Integrating cell-signalling pathways with NF-κB and IKK function**. *Nat Rev Mol Cell Biol* 2007, **8**(1):49-62.
14. Geva-Zatorsky N, Rosenfeld N, Itzkovitz S, Milo R, Sigal A, Dekel E, Yarnitzky T, Liron Y, Polak P, Lahav G *et al*: **Oscillations and variability in the p53 system**. *Mol Syst Biol* 2006, **2**:2006 0033.
15. Gillespie DT: **A General Method for Numerically Simulating the Stochastic Time Evolution of Coupled Chemical Reactions**. *Journal of Computational Physics* 1976, **22**:403-434.
16. Kreyzig E: **Advanced Engineering Mathematics**, 8th edn: John Wiley & Sons; 2005.
17. Lin CC, Segel LA: **Mathematics Applied to Deterministic Problems in the Natural Sciences** Society for Industrial and Applied Mathematics; 1988.
18. Tyson JJ, Novak B: **Regulation of the Eukariotic Cell Cycle: Molecular Antagonism, Hysteresis and Irreversible Transitions**. *Journal of Theoretical Biology* 2001, **210**:249-263.
